# The health impact of Scotland's Baby Box Scheme: a natural experiment evaluation using national linked health data

**DOI:** 10.1016/S2468-2667(23)00121-4

**Published:** 2023-06-29

**Authors:** Ronan McCabe, Srinivasa Vittal Katikireddi, Ruth Dundas, Peter Craig

**Affiliations:** a MRC/CSO Social and Public Health Sciences Unit, University of Glasgow, Glasgow, UK

## Abstract

**Background:**

Scotland's Baby Box Scheme (SBBS) is a national programme offering a box of essential items to all pregnant women in Scotland intended to improve infant and maternal health. We aimed to evaluate the effect of SBBS on selected infant and maternal health outcomes at population and subgroup levels (maternal age and area deprivation).

**Methods:**

Our complete-case, intention-to-treat evaluation used national health data (from the Scottish Morbidity Record [SMR] 01, SMR02, and the Child Health Surveillance Programme-Pre School), linking birth records to postnatal hospitalisation and universal health visitor records in Scotland. We considered maternal–infant pairs of all live-singleton births 2 years either side of SBBS introduction (Aug 17, 2015, to Aug 11, 2019). We estimated step-changes and trend-changes in outcomes (hospital admission and self-reported exclusive breastfeeding, tobacco smoke exposure, and infant sleeping position) by week of birth using segmented Poisson regression, adjusting for over-dispersion and seasonality where necessary.

**Findings:**

The analysis comprised 182 122 maternal–infant pairs. The prevalence of tobacco smoke exposure reduced after SBBS introduction: step decrease of 10% (prevalence ratio 0·904 [95% CI 0·865–0·946]; absolute decrease of 1·6% 1 month post-introduction) for infants and 9% (0·905 [0·862–0·950]; absolute decrease of 1·9% 1 month post-introduction) for the primary carer. There was no evidence of changes in infant and maternal all-cause hospital admissions or infant sleeping position. Among mothers younger than 25 years, there was a 10% step-increase in breastfeeding prevalence (1·095 [1·004–1·195]; absolute increase of 2·2% 1 month post-introduction) at 10 days and 17% (1·174 [1·037–1·328]) at 6–8 weeks postnatal. Although associations were robust to most sensitivity analyses, for smoke exposure associations were only observed early in the postnatal period.

**Interpretation:**

SBBS reduced infant and primary carer tobacco smoke exposure, and increased breastfeeding among young mothers in Scotland. However, absolute effects were small.

**Funding:**

Medical Research Council, Scottish Government Chief Scientist Office, and National Records of Scotland.

## Introduction

The early years of life are thought to constitute a crucial period, influencing health and health inequalities over the life-course and across generations.^1^, ^2^ For example, early life stress has been associated with heart disease, respiratory disease, alcohol and substance misuse, poor self-rated health, and inter-personal and self-directed violence in later life.^3^ Intervening at this early stage in life is thus a societal priority as recognised, for example, by the WHO Europe's child and adolescent health strategy and the Scottish Government's early years framework.^4^, ^5^ However, to ensure effective decision making and the appropriate targeting of resources, robust evaluation is necessary.^6^ Previous evaluations have examined, for example, the effect of the Health in Pregnancy Grant on birthweight in Scotland and the effect of child poverty on infant mortality in England.^7^, ^8^

Baby box schemes are a group of interventions providing parents with a cardboard box containing infant care supplies.^9^ These schemes are an example of non-monetary (or in-kind) transfers; however, the schemes differ in the care supplies provided and in their operation.^9^ These schemes have seen increasing international uptake; however, evidence of their effect on infant and maternal health is currently limited.^10^, ^11^ For example, a 2021 study evaluated an intervention involving a baby box, which aimed to reduce postnatal depression.^11^ However, the comparator population was not matched to the exposed population on characteristics relevant to the outcome and therefore the authors were unable to isolate intervention effects. Other research on baby boxes has focused on parental experiences and perceptions.^9^, ^12^, ^13^, ^14^ It is thus necessary to establish the health effect of these schemes.

Scotland's Baby Box Scheme (SBBS), alongside the original Finnish Maternity Package, are currently the only government-administered and non-commercial baby box schemes in operation. SBBS was introduced by the Scottish Government and has been available to all parents in Scotland since the Aug 15, 2017. The scheme involves a cardboard box containing various items that is fitted with a foam mattress intended for the infant to sleep in and is valued at approximately £298.^15^ Information on breastfeeding and safe sleeping practices (eg, infant sleeping position and tobacco smoke exposure) are provided within the box and on the Parent Club website associated with the scheme. Parents register for the scheme during the 20–24th week antenatal appointment and receive it between the 32nd and 36th week of pregnancy. Specified objectives of SBBS included increasing positive health behaviours, reducing risk health behaviours, and improving and reducing inequalities in infant and maternal health and wellbeing outcomes. A full description of SBBS following the Template for Intervention Description and Replication-Population Health and Policy is available in the appendix (p 2).^16^ We aimed to evaluate the effect of SBBS on selected maternal and infant health outcomes and investigate whether these effects differed by area deprivation and maternal age.

**Research in context**

**Evidence before this study**

We searched MEDLINE and SCOPUS for publications in English from database inception up to Nov 16, 2022, using the terms “baby box”, “baby kit”, “baby package”, “infant package”, and “matern* package”. Quantitative evidence on baby boxes and related interventions has been very scarce and there have been no randomised controlled trials to date. In a natural experiment evaluation, the effect of the Finnish Maternity Grant, which includes the Finnish Baby Box, on infant mortality was evaluated. However, the authors could not isolate intervention effects due to bias. Qualitative evidence on baby boxes has predominantly focused on parental perceptions. A qualitative study of parents receiving Scotland's Baby Box scheme (SBBS) found that perceptions were mostly positive; although, parents were more concerned with the social and practical implications of the scheme than its health impact.

**Added value of this study**

This is the first study to evaluate the health impact of a baby box scheme (SBBS) using individual-level linked health data and robust natural experimental methods. We included births occurring 2 years before the introduction of SBBS and 2 years following, achieving near complete population coverage. We found that SBBS reduced infant and primary carer tobacco smoke exposure. Although these effects were only present at 10 days postnatal, it should be noted that missingness was relatively high for measures further into the postnatal period. Stark differences were present by area deprivation for measures of tobacco smoke exposure, with the most deprived exhibiting the highest prevalence. However, there was no indication that SBBS narrowed these differences. SBBS had no effect on infant or maternal all-cause hospital admissions or infant sleeping position. SBBS did not affect exclusive breastfeeding at the population level but did increase exclusive breastfeeding among young mothers (aged <25 years). This effect persisted 6–8-weeks into the postnatal period and might have a beneficial impact on age-related inequalities, with young mothers exhibiting the lowest prevalence of exclusive breastfeeding.

**Implications of all the available evidence**

To date there has been very little evidence on the health effect of baby boxes and related interventions, despite increasing international uptake. Although we show a small beneficial effect for certain outcomes, further research is needed to establish intervention mechanisms and strengthen causal conclusions. More generally, the early years of life are crucial to health and development across the life-course. As such, the design of early years interventions should consider the determinants of infant health and incorporate outcome evaluation.

## Methods

### Study design

The introduction of SBBS on Aug 15, 2017, was evaluated as a natural experiment event using an intention-to-treat interrupted time series analysis. This analysis compared the post-intervention trend for each outcome with a counterfactual scenario formed through an extrapolation of the pre-intervention trend into the post-intervention period.^17^ We examined the impact of SBBS introduction in terms of both the step-change and slope-change in trend for outcomes relating to all-cause infant and maternal hospital admissions, infant and primary carer exposure to tobacco smoke, exclusive breastfeeding, and infant sleeping position.

### Data sources

We used three data sources: Scottish Morbidity Record (SMR) 01, SMR02, and the Child Health Surveillance Programme-Pre School (CHSP-PS). After initially identifying the study population in SMR02, extracts from these sources were deterministically linked via the Community Health Index. Linkage and analysis of these de-identified individual-level data was approved by the Public Benefit and Privacy Panel Committee of Public Health Scotland and did not require further ethnical approval (appendix p 2). Data extracts were provided according to study specifications, including study population and timeframe.

SMR01 collects data on all general and acute inpatient and day-case hospital activity in Scotland and SMR02 collects obstetric data covering 98% of all pregnancies and births in Scotland; these data are regularly audited at source.^18^ CHSP-PS data are self-reported and collected during universally provided postnatal infant health reviews which occur at 10 days, 6–8 weeks, and 13–15 months postnatal for all infants in Scotland. We included CHSP-PS data from the 10-day and 6–8-week reviews which covered 97% and 92%, respectively, of births in Scotland in 2018 limited to patients who have not actively opted out of data use in public interest by National Health Service Scotland.^19^

### Study population and timeframe

We included live singleton births occurring in Scotland between Aug 17, 2015, and Aug 11, 2019. Births occurring during the week of SBBS introduction (Aug 14–20, 2017) were excluded to provide a clearer demarcation between pre-intervention and post-intervention periods. We pursued a complete-case approach when defining the study population and removed missing observations across all included SMR02 variables before linkage (appendix p 3).

### Outcomes and subgroups

The outcomes were all-cause infant and maternal hospital admissions, maternal smoking, exclusive breastfeeding, infant sleeping position, and infant and primary carer tobacco smoke exposure. Full outcome measures used in this evaluation and the number of observations used are in the appendix (pp 3–4). Outcomes were selected on the basis of (1) a logic model for the scheme published by the Scottish Government,^20^ (2) the availability of relevant data, and (3) data completeness; elements of the logic model addressed and not addressed by this evaluation is in the appendix (p 4). We were unable to include infant mortality as an outcome due to low case numbers. Subgroups were defined by maternal age (<25 years, 25–38 years, and >38 years) and 2016 Scottish Index of Multiple Deprivation (SIMD) quintiles (1=most deprived and 5=least deprived), an area-based measure of deprivation commonly used as an indicator of socioeconomic position.^21^ Missing observations were removed before each analysis (did not apply to SMR02 measures). Missingness was high (9–24%) for measures derived from the CHSP-PS 6–8-week extract (appendix pp 3–4). Although missingness was not associated with either maternal age or SIMD quintile, it was associated with the start of the study timeframe for measures relating to tobacco smoke exposure, so we conducted sensitivity analyses excluding observations from the first 25 weeks of the series (appendix p 5).

### Statistical analysis

We estimated the relative step-change and slope-change between SBBS introduction and our outcome measures using segmented Poisson regression (appendix p 5).^22^ Model parameters for measures of incidence rates were rate ratios (RRs) and prevalence were prevalence ratioβs (PRs). Robust standard errors were computed using sandwich estimation for PRs.^23^ All outcomes were aggregated by week of delivery to form a time series. Absolute change was given as the difference between counterfactual (baseline or β_1_ in the model) and factual (modelled) values at 1 month (week 4) and 6 months (week 24) post-introduction. We used quasi-Poisson models to adjust for over-dispersion and Fourier terms (two sin and cosine pairs within a 52-week period) to adjust for seasonality.^24^ All models were checked for over-dispersion by estimating the dispersion parameter (values >1 indicated over-dispersion) and for autocorrelation using autocorrelative and partial-autocorrelative function plots. There was no residual autocorrelation after adjusting for seasonality in any of the models. To examine differential effects, we quantified subgroup interactions as the ratio of RRs or PRs from any two levels for all possible combinations.^25^

### Sensitivity analyses

Time-varying bias is the main threat to interrupted time series analysis.^17^ In addition to seasonality described above, the co-occurrence of events relevant to the outcome are important to consider (ie, history bias). Alongside considering possible sources of such bias, we conducted temporal falsification analyses to assess whether associations were specific to the point of SBBS introduction (the point of exposure was re-assigned to 8, 16, 24, and 48 weeks pre-SBBS and post-SBBS introduction). Temporal falsification would be viewed as supporting the original association if effect size waned progressively with increasing distance pre-introduction and post-introduction. To understand whether high missingness at the start of the study timeframe affected model estimates for measures relating to tobacco smoke exposure, we excluded the initial 24 weeks of the pre-intervention series; this was done for the main analysis of primary carer tobacco smoke exposure at 6–8 weeks postnatal as prevalence reduced to 0 for several of these weeks due to missingness. We included negative control analyses for tobacco exposure. Such analyses use outcomes that are probably affected by similar sources of bias as the outcome of interest but are not influenced by the exposure of interest.^26^ We included two self-reported prenatal outcomes as negative controls (maternal smoking at booking and maternal smoking during pregnancy).

### Role of the funding source

The funders of the study had no role in study design, data collection, data analysis, data interpretation, or writing of the report.

## Results

182 122 maternal–infant pairs of all live-singleton births were included in the base study population with a final observation period of 207 weeks (104 pre-introduction and 103 post-introduction). Summary statistics for the base study population are in table 1 and the appendix (p 6). Autocorrelative function and partial-autocorrelative function plots for all main models are in the appendix (pp 22–29). Subgroup means, estimates and interactions are in the appendix (pp 7–8, 11–19).

**Table 1:** Characteristics for the main study population

	**Maternal–infant pairs (n=182 122)**
**Maternal age, years**	
0–17	1484 (0·8%)
18–24	31 638 (17·4%)
25–31	75 047 (41·2%)
32–38	63 085 (34·6%)
39–44	10 428 (5·7%)
45–58	440 (0·2%)
**SIMD quintile**	
1 (most deprived)	44 818 (24·6%)
2	38 584 (21·2%)
3	33 210 (18·2%)
4	34 052 (18·7%)
5 (least deprived)	31 458 (17·3%)
**Previous pregnancy**	
Yes	121 542 (66·7%)
No	60 580 (33·3%)

Data are n (%). SIMD=Scottish Index of Multiple Deprivation.

Associated step-changes and slope-changes in the incidence of infant or maternal hospital admissions at either 26 or 52 weeks follow up were either small and imprecise or indicative of a null effect (table 2). Clear differences by socioeconomic position were present for both infant and maternal admissions with higher rates among the most deprived groups (appendix p 9); however, there was no indication of differential effects on all-cause hospital admissions by area deprivation or maternal age.

**Table 2:** Estimated relative and absolute change in outcomes following SBBS introduction

		**Relative change**		Absolute change*	
		Step-change estimate (95% CI)	Slope-change estimate (95% CI)	1 month after SBBS introduction	6 months after SBBS introduction
All-cause hospital admissions, incidence rate × 1000					
	Maternal, 26 weeks postnatal†	1·001 (0·879–1·141)	1·002 (1·000–1·004)	0·0	0·1
	Maternal, 52 weeks postnatal†	1·064 (0·965–1·173)	1·001 (0·999–1·002)	0·2	0·2
	Infant, 26 weeks postnatal†‡	0·972 (0·913–1·035)	1·001 (1·000–1·002)	−0·3	−0·1
	Infant, 52 weeks postnatal†	0·999 (0·943–1·059)	1·000 (0·999–1·001)	−0·0	−0·1
Exposure to tobacco smoke prevalence					
	Infant, 10 days postnatal‡	0·904 (0·865–0·946)	0·998 (0·997–0·999)	−1·6	−2·3
	Infant, 6–8 weeks postnatal†‡	1·017 (0·940–1·099)	1·004 (1·002–1·005)	0·3	0·8
	Primary carer, 10 days postnatal‡	0·905 (0·862–0·950)	0·998 (0·997–0·999)	−1·9	−2·8
	Primary carer, 6–8 weeks postnatal†	0·959 (0·907–1·014)	1·000 (0·999–1·001)	−0·6	−0·8
Prenatal maternal smoking prevalence					
	At booking†‡	0·990 (0·939–1·045)	0·999 (0·998–1·000)	−0·2	−0·5
	During pregnancy†‡	1·060 (1·008–1·114)	0·998 (0·998–0·999)	0·8	0·3
Exclusive breastfeeding prevalence					
	10 days postnatal‡	1·004 (0·978–1·031)	1·000 (1·000–1·001)	0·2	0·3
	6–8 weeks postnatal‡	1·020 (0·982–1·058)	1·000 (0·999–1·000)	0·6	0·5
Infant supine sleeping position (6-8 weeks postnatal) prevalence		1·004 (1·000–1·010)	1·000 (1·000–1·000)	0·4	0·1
Negative control measures					
	Maternal prenatal smoking at booking prevalence†‡	0·990 (0·939–1·045)	0·999 (0·998–1·000)	−0·2	−0·5
	Maternal prenatal smoking during pregnancy prevalence†‡	1·060 (1·008–1·114)	0·998 (0·998–0·999)	0·8	0·3

Estimate parameter for relative changes is rate ratio for measures of incidence rate and prevalence ratio for measures prevalence. SBBS=Scotland's Baby Box Scheme.

* Difference between modelled and baseline values.

† Model adjusted for over dispersion.

‡ Model adjusted for seasonality.

**Figure 1:**
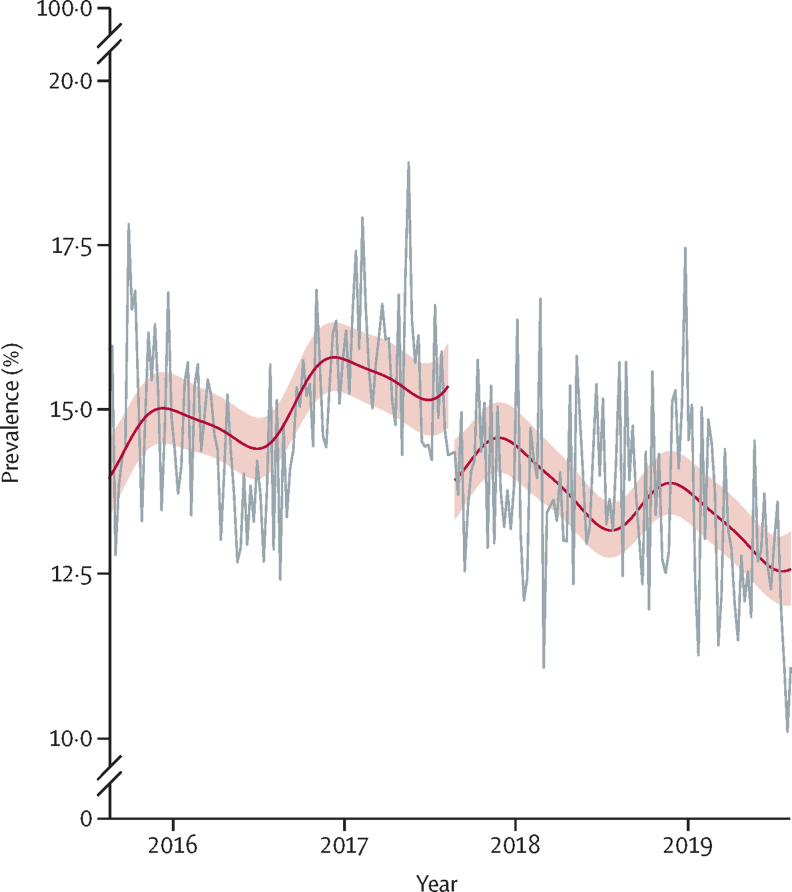
Prevalence of infant tobacco smoke exposure (grey horizontal), with modelled pre-introduction (left red horizontal) and post-introduction trends (right red horizontal) Shading represents 95% CIs for modelled values.

**Figure 2:**
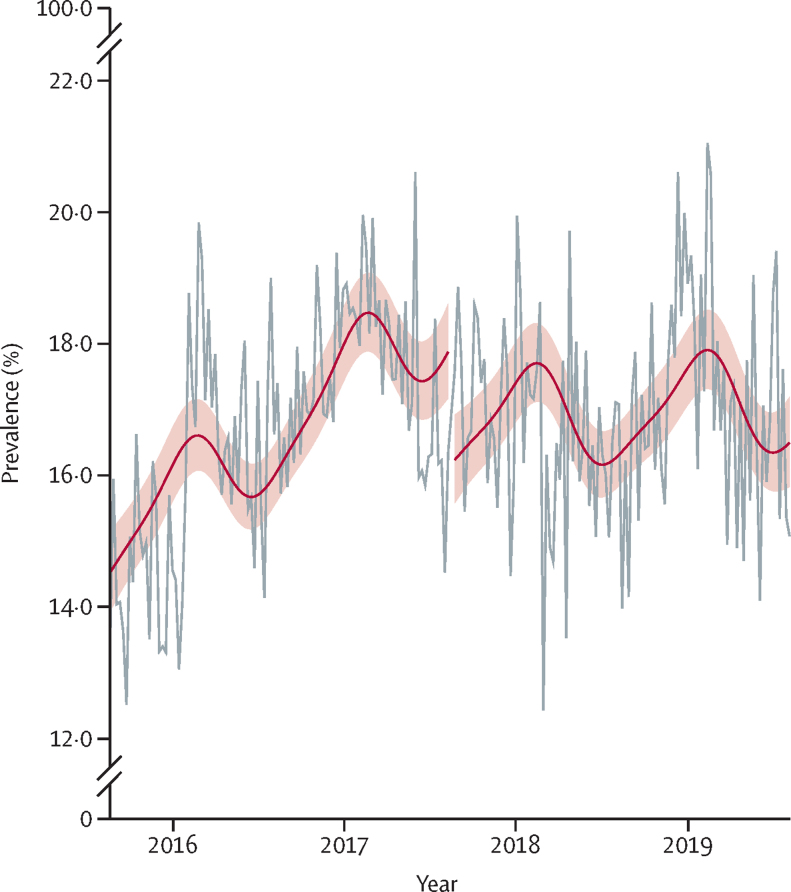
Prevalence of primary carers smoking (grey horizontal), with modelled pre-introduction and (left red horizontal) post-introduction trends (right red horizontal) Shading represents 95% CIs for modelled values.

SBBS was associated with a 10% step-decrease (PR 0·904 [95% CI 0·865–0·946]) and a 0·2% slope-decrease (0·998 [0·997–0·999]) in the prevalence of infants exposed to tobacco smoke, as measured at 10 days postnatal (table 2; figure 1). The association was robust to temporal falsification and truncation (appendix pp 13–20) and corresponds to an absolute decrease in prevalence of 1·6% at 1 month post-introduction and 2·3% at 6 months post-introduction. This association was not present at 6–8 weeks postnatal (1·017 [0·940–1·099; table 2). Similarly, SBBS was associated with a step-decrease in the prevalence of primary carers exposed to tobacco smoke at 10 days (0·905 [0·862–0·950]) but the size and precision of this estimate reduced at 6–8 weeks postnatal (0·959 [0·907–1·014]; table 2; figure 2). The change at 10 days postnatal corresponds to an absolute decrease of 1·9% at 1 month post-introduction and 2·8% 6 months post-introduction. This association was robust to truncation; however, temporal falsification did not clearly locate the association to the point of SBBS introduction (appendix pp 20–21). There was no decrease in prevalence for either negative control measures, suggesting this association was specific to the postnatal period (table 2); although, SBBS was associated with a 6% step-increase in smoking during pregnancy (1·060 [1·008–1·114]). Notable differences by socioeconomic position were observed for both infants and primary carers exposed to tobacco smoke, with prevalence of less than 5% among least deprived and more than 20% among most deprived (appendix pp 9–10). Subgroup analyses by deprivation did not indicate any clear differential effects; and an association similar in precision to the main analysis for infant exposure at 10 days postnatal was only present in the two most deprived groups (appendix pp 11–12), associations only significantly differed between SIMD quintile 2 (more deprived) and quintile 3 (appendix pp 13–16).

SBBS was not associated with any change in exclusive breastfeeding at 10 days postnatal (step PR 1·004 [95% CI 0·978–1·031]; slope 1·000 [1·000–1·001]) or 6–8 weeks postnatal (step 1·020 [0·982–1·058]; slope 1·000 [0·999–1·000]) at the population level (table 2). Clear differences were observed by age and socioeconomic position, with prevalence lowest among the youngest and most deprived groups (appendix p 10). Although there was no indication that SBBS narrowed differences by socioeconomic position in exclusive breastfeeding, the scheme was associated with a step-increase of 10% among young mothers (aged <25 years) 10 days postnatal (1·095 [1·004–1·195]) and 17% for 6–8 weeks postnatal (1·174 [1·037–1·329]; appendix pp 11–12). For 6–8 weeks postnatal, in absolute terms, this resembles an increase of 2·2% at 1 month post-introduction and 2·0% at 6 months post-introduction. These step-increases were significantly higher compared with older mothers (aged 25–38 years) at both 10 days (ratio of PRs 1·105 [95% CI 1·009–1·211]) and 6–8 weeks postnatal (1·175 [1·032–1·339]; appendix pp 17–19). Temporal falsification supported the association at 6–8 weeks postnatal but was more ambiguous at 10 days postnatal (appendix pp 20–21).

The prevalence of infant supine sleeping was high across the whole study timeframe (>90%; appendix p 10). There was no indication that the SBBS affected supine sleeping on the population level (step PR 1·004 [95% CI 1·000–1·010]; slope PR 1·000 [1·000–1·000]) or among subgroups (table 2). Unlike all other outcome measures, we did not observe any differences by socioeconomic position (appendix p 10).

## Discussion

SBBS was associated with a reduction in the proportion of infants exposed to second hand smoke, a reduction in primary carers smoking, and an increase in young mothers (aged <25 years) exclusively breastfeeding at 10 days postnatal. However, only the increase in breastfeeding persisted at 6–8 weeks. We observed no associated change in infant or maternal hospital admissions, exclusive breastfeeding on the population level, or infant sleeping position. Our findings imply a reduction of inequalities in exclusive breastfeeding by maternal age, with young mothers exhibiting a significant increase compared with older mothers.

We used robust methods of causal inference and benefited from near complete population coverage. The use of individual-level linked data allowed us to clearly define the study population, treatment groups, and sub-groups. However, we were unable to include sub-groups by other relevant demographic characteristics such as ethnicity; although these data were available, high missingness prevented their inclusion in our evaluation. Unlike a previous quasi-experimental study which evaluated the impact of the Finnish baby box scheme on infant mortality in the 20th century, we were able to isolate contemporary health effects across a range of outcomes.^10^ However, we could not include infant mortality, despite public interest in, which will require future research.

It is also worth considering potential sources of bias. Individuals who did not receive SBBS in the post-introduction period will have been misclassified by our intention-to-treat analysis. Uptake of SBBS was 85% of all new parents within the first year of introduction, rising to 96% in 2019 and 93% in 2020.^27^, ^28^, ^29^ The pilot scheme between January and March, 2017, is unlikely to constitute a major source of misclassification because only 160 boxes were distributed. Missingness was also relatively high for outcomes derived from the CHSP-PS 6–8-week postnatal extract; this was not clearly associated with either maternal age or SIMD quintile, and truncation was performed to exclude periods of high missingness at the start of the study timeframe. As a consequence of only using hospital admissions data, we were unable to capture any health effects occurring in other settings (eg, primary care); relatedly, it is possible these data capture health seeking behaviour rather than adverse events, however due to the relatively high threshold for hospital admission this is less likely.

Associations with infant and primary carer tobacco smoke exposure were robust to negative control analyses and, for infant exposure, temporal falsification. We deemed an associated step-increase for smoking during pregnancy to be spurious and unlikely to be indicative of bias because it was in the opposite direction to associations for our main measures of smoke exposure and was not present in the other negative control measure. There were no major fluctuations in tobacco price across the study timeframe and the standardisation of tobacco packaging in the UK between 2016 and 2017 (a relevant co-intervention) was a phased policy, so unlikely to produce the step-changes we observed.^30^ On the whole, the associations observed resemble a beneficial effect of SBBS early in the postnatal period. However, as our measures are self-reported, a more clinical measure such as infant hospital admissions for asthma and respiratory tract infection, which is responsive to tobacco control policy, would be worthy of future research.^31^ Nonetheless, because both parents and those responsible for data collection were masked to this evaluation, we do not consider reporting bias to be a major threat for any of the self-reported measures used. Relatedly, social desirability bias might be present to some extent in our self-reported measures; however, because SBBS involved minimal explicit health promotion, we think this is unlikely to be a major source of bias.

The increase in exclusive breastfeeding among young mothers was robust to temporal falsification at 6–8 weeks but not 10 days postnatal. We did not account for £2 million breastfeeding funding distributed by the Scottish Government in July 2018.^32^ However, this funding was almost a year after SBBS introduction, unlikely to benefit only young mothers, and was not evident from temporal falsification analyses. The Family Nurse Partnership is another relevant co-intervention because it provides young mothers in Scotland with regular support (care, advice, and information); however, most Scottish health boards have operated the scheme since 2015 (representing a majority of the population) making it unlikely a source of bias.

SBBS appears to have reduced infant and primary carer tobacco smoke exposure. However, these effects were small in absolute terms and only observed early in the postnatal period. We also observed a possible beneficial effect of SBBS on exclusive breastfeeding among young mothers, although also small in absolute terms. Mechanisms mediating these effects (eg, material effects, health information, or improved engagement with health care practitioners) require further investigation. We did not detect any effect on infant and maternal hospital admissions, exclusive breastfeeding on the population level, or infant sleeping position. Outcome evaluations of this kind are relatively inexpensive and straightforward and thus should be incorporated into the design of early years interventions when a randomised trial is not feasible. As others have noted, alongside outcome evaluations, understanding cost-effectiveness and the user experience are necessary to understand the wider social effect of SBBS and related schemes.^14^

## Data sharing

The data used for this study are retained by electronic Data Research and Innovation Service (eDRIS) at Public Health Scotland and are not publicly available. The data used for this study can be accessed through successful application to the NHS Scotland Public Benefit and Privacy Panel for Health and Social Care via eDRIS at https://www.informationgovernance.scot.nhs.uk/pbpphsc/. Code for analysis is available at https://github.com/MacCaibe/Scotlands-Baby-Box-Scheme-Evaluation.

## Declaration of interests

We declare no competing interests.
