# Supplementary material for: The health impact of Scotland's Baby Box Scheme: a natural experiment evaluation using national linked health data
Source: Lancet Public Health. 2023 Jun 29;8(7):e504–10. doi: 10.1016/S2468-2667(23)00121-4 (PMC10323308; doi:10.1016/S2468-2667(23)00121-4)
Supplement: Supplementary appendix [file mmc1.pdf]

THE LANCET
Public Health

Supplementary appendix

This appendix formed part of the original submission and has been peer reviewed. We post it as supplied by the authors.

Supplement to: McCabe R, Katikireddi SV, Dundas R, Craig P. The health impact of Scotland's Baby Box Scheme: a natural experiment evaluation using national linked health data. *Lancet Public Health* 2023; **8:** e504–10.

SUPPLEMENTAL MATERIAL

Contents

Appendix 1..2
Appendix 2..2
Appendix 3..3
Appendix 4..3
Appendix 5..4
Appendix 6..5
Appendix 7..5
Appendix 8..6
Appendix 9..7
Appendix 10..9
Appendix 11..11
Appendix 12..13
Appendix 13..20
Appendix 14..20
Appendix 15..22

Appendix 1

Guideline item	Description
1. Brief name	Scotland's Baby Box Scheme (or SBBS)
2. Why	The Scottish Government provided a logic model specifying the intended short-, medium-, and long-term objectives of the scheme. Of relevance to this evaluation, these included a) increased positive behaviours and reduced risk behaviours, b) improved infant/maternal health and wellbeing outcomes and c) reduced inequalities in infant/maternal health and wellbeing outcomes.
3. What materials	SBBS materially consists of a cardboard box containing various items 'essential' to the initial months of life. Box fitted with foam mattress and intended for infant sleeping. Information on breastfeeding, safe sleeping practices (e.g., risk factors such as non-supine sleeping and exposure to second-hand tobacco smoke), and postnatal depression contained within box and on associated *www.parentclub.scot* website. Parents are directed to website upon registration with SBBS and can opt-in to receive regular emails which deliver the information associated with the scheme (the frequency of emails, however, is not clear).
4. What & how	Initial registration in preparation for introduction began on June 15th 2017. Registration assisted by midwives and occurs during mothers 20–24th week antenatal appointment. Delivered between the 32nd and 36th week of pregnancy. Can be cancelled at any point prior to receipt.
5. Who provided	APS Group (Scotland) were commissioned by the Scottish Government to supply SBBS until July 2021 in a contract valued at £35.3 million. Each box, including contents and distribution, cost the Scottish Government approximately £160. The value of the box to parents was estimated to be approximately £298.
6. Where	Universally available to all mothers and new-born infants in Scotland.<br><br>There has been a longstanding emphasis on the early years in Scotland with the Getting it Right for Every Child approach and the Early Years Framework published by the Scottish Executive/Government in 2006 and 2008, respectively. Relevant to SBBS, the latter touches on the idea of 'critical periods', inequalities in health outcomes and the role of universal services targetting the early years. Scotland exhibits socio-economic inequalities across a range of early years health indicators including rates of exclusive breastfeeding, tobacco smoke exposure, premature birth, low birthweight and perinatal/infant mortality, with the most deprived groups exhibiting the worst outcomes. Of note, as a general indicator of infant health, infant mortality rates amongst the most deprived groups in Scotland increased between 2016 and 2018, despite reductions in the years preceding (2000 to 2018).
7. When & how often	Introduced by the Scottish Government on August 15th 2017. Pilot scheme was conducted between January and March 2017 in the local authority areas of Clackmannanshire and Orkney, involving the distribution of 160 boxes. Scheme is ongoing as of October 2022.
8. Planned/Unplanned variation	No planned or unplanned variation as of October 2022.
9. How well	Uptake was estimated by the Scottish Government at 85% of all new parents in first year of introduction, rising to 96% in 2019 and 93% in 2020. Registration data obtained by Bardsley et al. (2021) shows that uptake did not differ meaningfully by area level deprivation.

Description of SBBS following TIDieR-PHP template. SBBS is described here using an extension of the Template for Intervention Description and Replication adapted to Population Health and Policy interventions (TIDieR-PHP).

Appendix 2

All data used in this study were collected by National Health Service (NHS) Scotland in line with their statutory duties and service provision (https://www.nhsinform.scot/care-support-and-rights/health-rights/confidentiality-and-data-protection/how-the-nhs-handles-your-personal-health-information). This is communicated to patients through Data Protection Notices, information leaflets and discussions with care providers. All patients have the right to refuse use of their data by NHS Scotland through active opt-out. Permission to use the data in this study was granted via successful application to Public Health Scotland's (part of NHS Scotland) Public Benefit and Privacy Panel for Health and Social Care (https://www.informationgovernance.scot.nhs.uk/pbpphsc/). The use of these secondary data were covered by generic ethical approval granted to Public Health Scotland (https://www.researchdata.scot/ethics-other-approvals).

Appendix 3

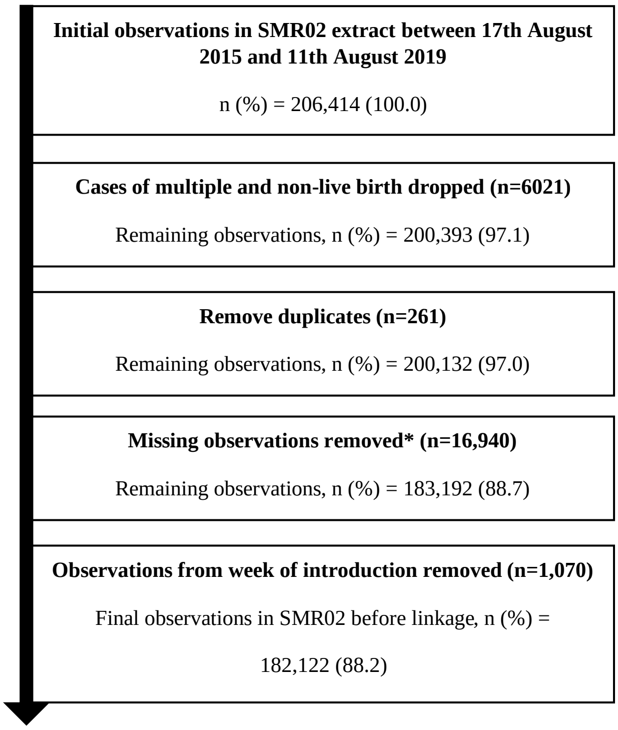

Data processing for SMR-02 extract, used as population spine. *missing observations were removed across the following variables (n and % missing in SMR02 extract): sex (91; 0.05), Feed on discharge (6,230; 3.11), Total previous pregnancies (1,034; 0.52), Smoking history at booking (6,441; 3.22), Smoking during pregnancy (8,727; 4.36), Mothers age (0; 0), SIMD-2016 (346; 0.17).

Appendix 4

Outcome	Source	Observations used in analysis (% missing)
All-cause maternal hospital admissions (26 weeks postnatal), incidence rate x 1000	SMR01	182,122 (0)
All-cause maternal hospital admissions (52 weeks postnatal), incidence rate x 1000		182,122 (0)
All-cause infant hospital admissions (26 weeks postnatal), incidence rate x 1000		182,122 (0)
All-cause infant hospital admissions (52 weeks postnatal), incidence rate x 1000		182,122 (0)
Maternal smoking at booking (prenatal), prevalence	SMR02	182,122 (0)
Maternal smoking during pregnancy (prenatal), prevalence		182,122 (0)
Exclusive breastfeeding (10-days postnatal), prevalence	CHSP-PS	178,546 (2·0)
Exclusive breastfeeding (6-8 weeks postnatal), prevalence		165,189 (9·3)
Infant sleeping position (6-8-weeks postnatal), prevalence		161,108 (11·5)
Infant tobacco smoke exposure (10-days postnatal), prevalence		176,864 (2·9)
Infant tobacco smoke exposure (6-8 weeks postnatal), prevalence		153,453 (15·7)
Primary carer tobacco smoke exposure (10-days postnatal), prevalence		177,363 (2·6)
Primary carer tobacco smoke exposure (6-8 weeks postnatal), prevalence		139,185 (23·6)

Outcomes, data sources and observations used in analysis. Provides the number of observations used and the percentage (%) missing for each outcome measure used in the evaluation. Note, this table represents the complete-case SMR02 extract used as the population spine; for level of missingness in SMR02 variables prior to forming complete-case extract see Appendix 3.

Appendix 5

Policy outcomes from Evaluability Assessment logic model	Reason for inclusion/exclusion in evaluation
Shared understanding of a society that values and supports all children.	Excluded as does not immediately concern health and not measurable with health records.
Reduced expenditure on new-born essentials AND *Reduced inequalities in access to new-born essentials*	Excluded as does not immediately concern health and not measurable with health records.
Parents understand and use box and its contents.	Excluded as does not immediately concern health and not measurable with health records.
Increased parent and workforce understanding of risk and positive behaviours (breastfeeding, sleeping practice, health seeking behaviour and play/attachment).	Excluded as not directly measurable with health records. More amenable to survey and qualitative methods.
Increased positive behaviours and decreased risk behaviours	Included in our evaluation with measures of breastfeeding, sleeping position and tobacco smoke exposure. Data on play/attachment was not available to us. We intended to model health seeking behaviour using infant immunisation uptake, however unresolved data quality issues led to outcome being dropped from evaluation.
Workforce understands contents and purpose of box	Excluded as not directly measurable with health records. More amenable to survey and qualitative methods.
[parents] Attempt to engage with wider services AND *Sustained engagement with wider services*	Intended to be included with infant immunisation uptake used as a proxy measure of service engagement, however unresolved data quality issues led to outcome being dropped from evaluation.
Improved maternal/child health & wellbeing outcomes	Included with measures of infant and maternal hospital admissions. Evaluation initially intended to use prescriptions data for anxiolytic and anti-depressant medication to understand possible changes in maternal mental health, however these data were not received.
Reduced inequalities in maternal/child health & wellbeing outcomes	Included with sub-group analyses by area-level deprivation and maternal age for all outcome measures in evaluation.
Reduced inequalities and improvement in early years development outcomes.	Intended to be included using measures of infant development from the CHSP-PS data extracts. However, these measures were excluded on the basis of high missingness.
Reduced infant mortality	Excluded due to lack of adequate statistical power; infant mortality rates at the time of SBBS introduction were ~4 deaths per 1000 live births, which resembles the weekly rate with ~50,000 births occurring annually in Scotland.

Scotland's Baby Box Scheme (SBBS) policy outcomes and reasons for inclusion/exclusion in present evaluation. These outcomes were outlined by a logic model in an Evaluability Assessment for SBBS that was commissioned by the Scottish Government: https://www.gov.scot/publications/evaluability-assessment-scotlands-baby-box-report-scottish-government/documents/

Appendix 6

Variable	Levels	n missing (%)		
		**Exclusive breastfeeding (6-8 weeks postnatal)**	**Primary carer smoking (6-8 weeks postnatal)**	**Infant exposure to tobacco smoke (6-8 weeks postnatal)**
Maternal Age (years)	0-17	222 (15·0)	417 (28·1)	315 (21·2)
	18-24	3430 (10·8)	7404 (23·4)	5440 (17·2)
	25-31	6761 (9·0)	15920 (21·2)	11631 (15·5)
	32-38	5558 (8·8)	12972 (20·6)	9649 (15·3)
	39-44	915 (8·8)	2085 (20·0)	1566 (15·0)
	45-58	47 (10·7)	102 (23·2)	68 (15·5)
SIMD quintile	1 (most deprived)	4582 (10·2)	9622 (21·5)	6999 (15·6)
	2	3594 (9·3)	8394 (21·8)	6202 (16·1)
	3	2973 (9·0)	7047 (21·2)	5191 (15·6)
	4	3016 (8·9)	7179 (21·1)	5446 (16·0)
	5 (least deprived)	2768 (8·8)	6658 (21·2)	4831 (15·4)
Previous pregnancy	Yes	11293 (9·3)	26074 (21·5)	19160 (15·8)
	No	5640 (9·3)	12826 (21·2)	9509 (15·7)

Level of missing by population characteristic for selected outcomes. This table provides the level of missingness by population characteristics for three outcomes derived from the CHSP-PS 6-8-week review data extract.

Appendix 7

Segmented regression model used in evaluation:

$$\log(Y_t/B_t) = \beta_0 + \beta_1 \cdot time_t + \beta_2 \cdot step_t + \beta_3 \cdot slope_t + e_t$$

Y_t represented the number of events at any given week of the study timeframe (t). For measures of hospital admission, B_t represents person-weeks at risk; for all other measures, B_t represents the number of observations at *t*. Y_t/B_t thus gives the person-week incidence rate for the former and prevalence for the latter. β_0 gives the y-intercept (t=0), β_1 estimates the baseline trend, β_2 estimates the step-change following SBBS introduction, and β_3 estimates the slope-change following SBBS introduction.

Appendix 8

Pre-introduction				Post-introduction			
**Min**	**Max**	**Median**	**IQR**	**Min**	**Max**	**Median**	**IQR**
All-cause maternal hospital admissions (26 weeks postnatal), incidence rate x 1000							
1·42	4·84	2·6	2·9-2·2	1·40	5·57	2·6	3·0-2·2
All-cause maternal hospital admissions (52 weeks postnatal), incidence rate x 1000							
1·6	4·3	2·6	2·9-2·4	4·5	1·5	2·8	3·0-2·5
All-cause infant hospital admissions (26 weeks postnatal), incidence rate x 1000							
6·7	13·3	10·19	11·1-9·2	8·7	16·7	11·4	12·3-10·4
All-cause infant hospital admissions (52 weeks postnatal), incidence rate x 1000							
5·9	9·7	7·7	8·2-7·1	6·4	11·9	8·2	8·9-7·7
Exclusive breastfeeding (10-days postnatal), prevalence							
31·4	42·2	37·0	38·2-35·5	32·8	41·1	38·1	39·2-36·4
Exclusive breastfeeding (6-8 weeks postnatal), prevalence							
26·1	35·5	30·5	31·6-28·9	27·8	36·7	32·4	33·5-30·8
Infant sleeping position (6-8 days postnatal), prevalence							
90·3	95·8	93·1	93·9-92·5	91·4	97·3	94·7	95·4-94·0
Infant exposure to tobacco smoke (10-days postnatal), prevalence							
12·4	18·7	15·1	15·9-14·3	10·1	17·4	13·6	14·5-12·8
Infant exposure to tobacco smoke (6-8 weeks postnatal), prevalence							
6·5	14·8	9·3	10·8-8·4	5·6	10·9	7·9	8·53-7·18
Primary carer smoking (10-days postnatal), prevalence							
12·4	18·7	16·8	18·0-15·7	10·1	17·4	17·0	17·8-15·9
Primary carer smoking (6-8 weeks postnatal), prevalence							
11·3	17·4	14·2	15·2-13·2	10·4	17·3	14·1	15·0-13·2
Maternal smoking at booking (prenatal), prevalence							
12·0	18·9	15·0	16·1-14·2	11·3	18·4	14·4	15·6-13·5
Maternal smoking during pregnancy (prenatal), prevalence							
12·6	20·3	16·0	17·0-15·1	13·5	21·9	17·1	18·5-16·1

Summary statistics for main outcome measures used in the evaluation. This table provides minimum, maximum, median values and the inter-quartile rang (IQR) for pre- and post-intervention periods for each outcome measure.

Appendix 9

Outcome	Subgroup mean pre- and post-SBBS introduction															
	Maternal age (years)						*SIMD quintile*									
	<25		*25-38*		*>38*		*1 (most deprived)*		*2*		*3*		*4*		*5 (least deprived)*	
	Pre	*Post*	*Pre*	*Post*	*Pre*	*Post*	*Pre*	*Post*	*Pre*	*Post*	*Pre*	*Post*	*Pre*	*Post*	*Pre*	*Post*
All-cause maternal hospital admissions (26 weeks postnatal), incidence rate x 1000	3.65	3.91	2.38	2.34	2.39	2.71	3.57	3.45	2.84	3.10	2.37	2.33	2.12	2.03	1.79	1.94
All-cause maternal hospital admissions (52 weeks postnatal), incidence rate x 1000	3.80	4.27	2.45	2.47	2.24	2.59	3.61	3.70	2.97	3.24	2.47	2.52	2.24	2.17	1.77	1.93
All-cause infant hospital admissions (26 weeks postnatal), incidence rate x 1000	13.07	14.92	9.50	10.91	8.67	9.54	11.10	13.09	10.78	12.69	9.99	10.86	9.55	10.78	8.71	9.42
All-cause infant hospital admissions (52 weeks postnatal), incidence rate x 1000	10.01	10.92	7.18	7.92	6.43	6.72	8.54	9.63	8.29	9.17	7.47	7.98	7.05	7.81	6.53	6.66

Exclusive breastfeeding (10-days postnatal), prevalence	18.97	20.87	41.02	40.87	45.49	45.12	22.93	23.66	29.39	30.26	39.21	40.24	46.83	47.12	53.20	54.00
Infant sleeping position (6-8 days postnatal), prevalence	92.79	94.66	93.15	94.77	93.30	95.45	93.28	94.48	92.88	94.51	92.85	94.63	93.08	94.68	93.31	95.30
Infant exposure to tobacco smoke (10-days postnatal), prevalence	27.69	24.78	12.24	11.47	10.47	8.97	24.78	22.64	19.16	17.55	13.14	12.37	8.43	7.64	4.91	4.19
Primary carer smoking (10-days postnatal), prevalence	29.99	30.66	13.75	14.34	10.99	10.59	28.39	28.91	21.31	21.54	14.37	15.03	8.83	9.31	4.81	4.89
Maternal smoking at booking (prenatal), prevalence	28.34	28.32	12.16	12.00	9.11	8.24	26.71	26.73	19.12	18.85	12.71	11.87	7.61	7.29	3.60	3.36
Maternal smoking during pregnancy (prenatal), prevalence	29.50	31.90	13.12	14.52	9.73	10.03	27.38	29.45	19.80	21.57	13.68	14.83	9.03	9.61	4.95	5.89

Subgroup means across pre- and post-SBBS periods for given outcome measures. This table does not include breastfeeding and smoking measures derived from the CHSP-PS 6-8 week extract as these were not extracted for publication prior to the expiration of data access permissions.

Appendix 10

SIMD quintile	Mean incidence rate x 1000			
	Pre-introduction	Post-introduction	Difference (post – pre)	Ratio (post / pre)
1 (most deprived)	11·10	13·09	2	1·2
2	10·78	12·69	1·9	1·2
3	9·99	10·86	0·9	1·1
4	9·55	10·78	1·2	1·1
5 (least deprived)	8·71	9·42	0·7	1·1

A. Comparison of mean incidence rate (x 1000) of all-cause infant hospital admissions at 26 weeks follow-up between pre- and post-introduction periods by Scottish Index of Multiple Deprivation (SIMD) quintile.

SIMD quintile	Mean incidence rate x 1000			
	Pre-introduction	Post-introduction	Difference (post – pre)	Ratio (post / pre)
1 (most deprived)	3·6	3·5	-0·1	1
2	2·8	3·1	0·3	1·1
3	2·4	2·3	-0·1	1
4	2·1	2·0	-0·1	1
5 (least deprived)	1·8	1·9	0·1	1·1

B. Comparison of mean incidence rate (x 1000) of all-cause maternal hospital admissions at 26 weeks follow-up between pre- and post-introduction periods by Scottish Index of Multiple Deprivation (SIMD) quintile.

SIMD quintile	Mean prevalence (%)			
	Pre-introduction	Post-introduction	Difference (post - pre)	Ratio (post / pre)
1 (most deprived)	24·8	22·6	-2·2	0·9
2	19·2	17·6	-1·6	0·9
3	13·1	12·4	-0·7	1
4	8·4	7·6	-0·8	0·9
5 (least deprived)	4·9	4·2	-0·7	0·9

C. Comparison of mean prevalence of infant exposure to tobacco smoke (10-days postnatal) between pre- and post-introduction periods by Scottish Index of Multiple Deprivation (SIMD) quintile.

SIMD quintile	Mean prevalence (%)			
	Pre-introduction	Post-introduction	Difference (post – pre)	Ratio (post / pre)
1 (most deprived)	28·4	28·9	0·5	1
2	21·3	21·5	0·2	1
3	14·4	15·0	0·6	1
4	8·8	9·3	0·5	1·1
5 (least deprived)	4·8	4·9	0·1	1

D. Comparison of mean prevalence of primary carers smoking (10-days postnatal) between pre- and post-introduction periods by Scottish Index of Multiple Deprivation (SIMD) quintile.

SIMD quintile	Mean prevalence (%)			
	Pre-introduction	Post-introduction	Difference (post – pre)	Ratio (post / pre)
1 (most deprived)	22·93	23·66	0·7	1
2	29·39	30·26	0·9	1
3	39·21	40·24	1	1
4	46·83	47·12	0·3	1
5 (least deprived)	53·2	54·0	0·8	1

E. Comparison of mean prevalence of exclusive breastfeeding (10-days postnatal) between pre- and post-introduction periods by Scottish Index of Multiple Deprivation (SIMD) quintile.

Maternal age (years)	Mean prevalence (%)			
	Pre-introduction	Post-introduction	Difference (post – pre)	Ratio (post / pre)
<25	12·9	14·8	1·9	1·1
25-38	34·1	35·4	1·3	1
>38	39·3	40·6	1·3	1

F. Comparison of mean prevalence of exclusive breastfeeding (10-days postnatal) between pre- and post-introduction periods by maternal age group.

SIMD quintile	Mean prevalence (%)			
	Pre-introduction	Post-introduction	Difference (post – pre)	Ratio (post / pre)
1 (most deprived)	93·28	94·44	1·2	1
2	92·88	94·38	1·5	1
3	92·85	94·6	1·8	1
4	93·08	94·84	1·8	1
5 (least deprived)	93·31	94·97	1·7	1

G. Comparison of mean prevalence of supine sleeping (6-8-weeks postnatal) between pre- and post-introduction periods by Scottish Index of Multiple Deprivation (SIMD) quintile.

Appendix 11

Subgroup	Step-change estimate (95% CI)	Slope-change estimate (95% CI)
All-cause maternal hospital admissions (26 weeks postnatal), incidence rate x 1000		
Maternal age (years)		
<25	1·1099 (0·8982, 1·3715)	1·0016 (0·9981, 1·0052)
25-38	0·9604 (0·8213, 1·1231)	1·0021 (0·9995, 1·0047)
>38	0·9812 (0·5530, 1·7409)	1·0059 (0·9967, 1·0152)
SIMD quintile		
1 (most deprived)	1·0387 (0·8532, 1·2646)	1·0028 (0·9996, 1·0061)
2	1·0427 (0·8256, 1·3167)	1·0011 (0·9972, 1·0049)
3	0·9596 (0·6858, 1·3428)	1·0030 (0·9975, 1·0085)
4	0·9360 (0·6954, 1·2598)	1·0012 (0·9962, 1·0061)
5 (least deprived)	0·9944 (0·6534, 1·5134)	1·0027 (0·9958, 1·0096)
All-cause maternal hospital admissions (52 weeks postnatal), incidence rate x 1000		
Maternal age (years)		
<25	1·1182 (0·9404, 1·3296)	1·0016 (0·9988, 1·0045)
25-38	1·0518 (0·9362, 1·1817)	0·9999 (0·9979, 1·0018)
>38	0·9078 (0·5601, 1·4714)	1·0054 (0·9976, 1·0133)
SIMD quintile		
1 (most deprived)	1·1060 (0·9652, 1·2672)	1·0005 (0·9983, 1·0028)
2	1·1246 (0·9285, 1·3621)	1·0015 (0·9984, 1·0047)
3	1·0271 (0·7958, 1·3257)	1·0011 (0·9968, 1·0053)
4	0·9938 (0·7931, 1·2453)	0·9988 (0·9950, 1·0026)
5 (least deprived)	1·0179 (0·7198, 1·4395)	1·0004 (0·9946, 1·0062)
All-cause infant hospital admissions (26 weeks postnatal), incidence rate x 1000		
Maternal age (years)		
<25 [s]	0·9626 (0·8489, 1·0916)	1·0011 (0·9991, 1·0030)
25-38 [s]	0·9715 (0·9026, 1·0457)	1·0008 (0·9997, 1·0019)
>38	1·0836 (0·8475, 1·3854)	0·9985 (0·9944, 1·0027)
SIMD quintile		
1 [s] (most deprived)	0·9818 (0·8762, 1·1001)	1·0008 (0·9991, 1·0026)
2 [s]	1·0085 (0·8874, 1·1461)	1·0003 (0·9984, 1·0023)
3	1·0000 (0·8749, 1·1429)	0·9996 (0·9973, 1·0018)
4	1·0832 (0·9417, 1·2459)	1·0012 (0·9989, 1·0036)
5 (least deprived)	1·0240 (0·8778, 1·1946)	1·0017 (0·9991, 1·0042)
All-cause infant hospital admissions (52 weeks postnatal), incidence rate x 1000		
Maternal age (years)		
<25	0·9753 (0·8732, 1·0895)	1·0000 (0·9981, 1·0018)
25-38	0·9992 (0·9357, 1·0670)	0·9996 (0·9985, 1·0007)
>38	1·0837 (0·8289, 1·4168)	0·9994 (0·9949, 1·0039)
SIMD quintile		
1 (most deprived)	0·9946 (0·9002, 1·0989)	0·9997 (0·9980, 1·0014)
2	1·0090 (0·9012, 1·1298)	0·9997 (0·9978, 1·0016)
3	0·9775 (0·8713, 1·0966)	0·9979 (0·9959, 0·9998)
4	1·0668 (0·9527, 1·1945)	1·0003 (0·9984, 1·0022)
5 (least deprived)	0·9523 (0·8219, 1·1032)	1·0009 (0·9985, 1·0033)
Exclusive breastfeeding (10-days postnatal), prevalence		
Maternal age (years)		
<25	1·0951 (1·0040, 1·1945)	0·9997 (0·9982, 1·0012)
25-38 [s]	0·9907 (0·9627, 1·0194)	1·0002 (0·9997, 1·0006)
>38	0·9923 (0·9108, 1·0810)	1·0003 (0·9989, 1·0018)
SIMD quintile		
1 (most deprived)	0·9923 (0·9350, 1·0533)	1·0002 (0·9992, 1·0013)
2	0·9418 (0·8834, 1·0039)	0·9997 (0·9986, 1·0008)
3	1·0250 (0·9753, 1·0773)	0·9998 (0·9989, 1·0006)
4	0·9861 (0·9409, 1·0335)	1·0005 (0·9997, 1·0012)
5 (least deprived)	0·9894 (0·9474, 1·0332)	1·0004 (0·9997, 1·0011)
Exclusive breastfeeding (6-8 weeks postnatal), prevalence		
Maternal age (years)		
<25	1·1739 (1·0374, 1·3285)	0·9992 (0·9971, 1·0013)
25-38 [s]	0·9989 (0·9592, 1·0403)	0·9999 (0·9993, 1·0005)
>38	1·0007 (0·8831, 1·1340)	0·9997 (0·9976, 1·0018)
SIMD quintile		
1 (most deprived)	1·0156 (0·9354, 1·1027)	1·0004 (0·9990, 1·0018)
2	0·9350 (0·8593, 1·0172)	0·9988 (0·9974, 1·0003)
3	1·0196 (0·9435, 1·1018)	0·9992 (0·9979, 1·0005)
4	0·9930 (0·9261, 1·0647)	1·0002 (0·9990, 1·0013)

5 (least deprived)	1·0284 (0·9555, 1·1068)	1·0002 (0·9991, 1·0013)
Infant sleeping position (6-8 weeks postnatal), prevalence		
Maternal age (years)		
<25	1·0059 (0·9939, 1·0180)	1·0000 (0·9998, 1·0002)
25-38 [s]	1·0010 (0·9946, 1·0074)	0·9998 (0·9997, 0·9999)
>38	0·9933 (0·9748, 1·0121)	0·9997 (0·9994, 1·0001)
SIMD quintile		
1 (most deprived)	1·0038 (0·9940, 1·0137)	0·9999 (0·9997, 1·0001)
2	1·0037 (0·9933, 1·0142)	0·9999 (0·9997, 1·0000)
3	1·0016 (0·9899, 1·0134)	0·9999 (0·9997, 1·0001)
4	1·0051 (0·9922, 1·0181)	0·9999 (0·9997, 1·0001)
5 (least deprived)	1·0083 (0·9964, 1·0205)	0·9998 (0·9996, 1·0000)
Infant tobacco smoke exposure (10-days postnatal), prevalence		
Maternal age (years)		
<25	0·9318 (0·8720, 0·9958)	0·9974 (0·9961, 0·9987)
25-38	0·9217 (0·8697, 0·9769)	0·9987 (0·9977, 0·9997)
>38	0·9060 (0·7234, 1·1347)	0·9968 (0·9928, 1·0008)
SIMD quintile		
1 (most deprived)	0·9155 (0·8569, 0·9781)	0·9981 (0·9969, 0·9993)
2	0·8978 (0·8306, 0·9705)	0·9986 (0·9971, 1·0000)
3	1·0337 (0·9285, 1·1507)	0·9973 (0·9954, 0·9992)
4 [d]	1·0258 (0·8800, 1·1957)	0·9991 (0·9964, 1·0018)
5 (least deprived)	0·8550 (0·6878, 1·0627)	0·9972 (0·9938, 1·0007)
Infant tobacco smoke exposure (6-8 weeks postnatal), prevalence		
Maternal age (years)		
<25	0·9891 (0·8813, 1·1098)	1·0019 (1·0000, 1·0039)
25-38 [d, s]	1·0194 (0·9267, 1·1214)	1·0047 (1·0032, 1·0062)
>38	1·4012 (0·9706, 2·0246)	1·0038 (0·9978, 1·0099)
SIMD quintile		
1 (most deprived)	1·0836 (0·9758, 1·2031)	1·0027 (1·0009, 1·0044)
2 [d]	0·9994 (0·8733, 1·1435)	1·0043 (1·0021, 1·0065)
3	1·1361 (0·9572, 1·3480)	1·0036 (1·0008, 1·0065)
4 [d]	1·0980 (0·8765, 1·3741)	1·0037 (1·0000, 1·0075)
5 [d] (least deprived)	0·9727 (0·7039, 1·3402)	1·0066 (1·0013, 1·0119)
Primary carer tobacco smoke exposure (10-days postnatal), prevalence		
Maternal age (years)		
<25	0·9637 (0·8932, 1·0398)	0·9982 (0·9969, 0·9995)
25-38 [s]	0·8768 (0·8287, 0·9277)	0·9982 (0·9972, 0·9991)
>38 [d]	0·8676 (0·6861, 1·0972)	0·9995 (0·9956, 1·0035)
SIMD quintile		
1 (most deprived)	0·9766 (0·9197, 1·0370)	0·9991 (0·9981, 1·0001)
2	0·9205 (0·8566, 0·9891)	0·9982 (0·9969, 0·9995)
3	0·8845 (0·7970, 0·9817)	0·9970 (0·9952, 0·9988)
4 [d]	0·9086 (0·7753, 1·0649)	0·9967 (0·9939, 0·9996)
5 (least deprived)	0·7981 (0·6549, 0·9725)	0·9982 (0·9950, 1·0015)
Primary carer tobacco smoke exposure (6-8 weeks postnatal), prevalence		
Maternal age (years)		
<25	1·0049 (0·9141, 1·1050)	0·9991 (0·9972, 1·0010)
25-38 [s]	0·9347 (0·8717, 1·0023)	1·0000 (0·9986, 1·0014)
>38	0·8630 (0·6440, 1·1579)	0·9996 (0·9938, 1·0054)
SIMD quintile		
1 (most deprived)	0·9774 (0·8989, 1·0629)	1·0000 (0·9983, 1·0016)
2	0·9666 (0·8685, 1·0761)	0·9991 (0·9970, 1·0013)
3	0·9604 (0·8368, 1·1026)	0·9991 (0·9964, 1·0019)
4 [d]	1·0451 (0·8600, 1·2713)	0·9994 (0·9955, 1·0033)
5 (least deprived)	0·7897 (0·6025, 1·0351)	1·0049 (0·9997, 1·0101)

Subgroup estimates with 95% CI for main outcome measures used in evaluation. Estimate parameter is Rate Ratio (RR) for measures of incidence rate and Prevalence Ratio (PR) for measures prevalence. [s] = model adjusted for seasonality, [d] = model adjusted for over-dispersion.

Appendix 12

	Step<br>Ratio of RRs/PRs (95% CI), *p-value*				Slope<br>Ratio of PRs/RRs (95% CI)			
**SIMD**	2	3	4	5<br>(least deprived)	2	3	4	5 (least deprived)
**All-cause maternal hospital admissions (26 weeks postnatal), incidence rate**								
1 (most deprived)	0.9962 (0.7341-1.3518), *0.98*	1.0824 (0.7334-1.5977), *0.7*	1.1097 (0.7771-1.5848), *0.58*	1.0445 (0.6569-1.6609), *0.86*	1.0017 (0.9967-1.0067), *0.52*	0.9998 (0.9935-1.0062), *0.96*	1.0016 (0.9957-1.0075), *0.61*	1.0001 (0.9925-1.0077), *0.98*
2		1.0866 (0.7218-1.6358), *0.7*	1.1140 (0.7635-1.6254), *0.59*	1.0486 (0.6485-1.6953), *0.86*	2	0.9981 (0.9914-1.0048), *0.59*	0.9999 (0.9937-1.0062), *0.98*	0.9984 (0.9906-1.0063), *0.7*
3			1.0252 (0.6547-1.6054), *0.92*	0.9650 (0.5636-1.6523), *0.9*	3		1.0018 (0.9944-1.0092), *0.65*	1.0003 (0.9915-1.0091), *0.95*
4				0.9413 (0.5627-1.5745), *0.83*	4			0.9985 (0.9901-1.0070), *0.74*
**All-cause maternal hospital admissions (52 weeks postnatal), incidence rate**								
1 (most deprived)	0.9835 (0.7775-1.2440), *0.9*	1.0768 (0.8064-1.4379), *0.63*	1.1129 (0.8551-1.4484), *0.43*	1.0866 (0.7488-1.5767), *0.68*	0.9990 (0.9951-1.0029), *0.63*	0.9994 (0.9946-1.0042), *0.82*	1.0017 (0.9973-1.0061), *0.46*	1.0001 (0.9939-1.0063), 0.98
2		1.0949 (0.7958-1.5065), *0.59*	1.1316 (0.8417-1.5214), *0.42*	1.1048 (0.7436-1.6416), *0.63*	2	1.0004 (0.9951-1.0057), *0.89*	1.0027 (0.9978-1.0077), *0.29*	1.0011 (0.9945-1.0077), *0.76*
3			1.0335 (0.7352-1.4529), *0.86*	1.0090 (0.6562-1.5517), *0.97*	3		1.0023 (0.9966-1.0080), *0.44*	1.0007 (0.9935-1.0079), *0.86*
4				0.9763 (0.6457-1.4763), *0.92*	4			0.9984 (0.9915-1.0053), *0.66*
**All-cause infant hospital admissions (26 weeks postnatal), incidence rate**								
1 (most deprived)	0.9735 (0.8203-1.1553), *0.77*	0.9818 (0.8238-1.1701), *0.85*	0.9064 (0.7568-1.0856), *0.29*	0.9588 (0.7917-1.1612), *0.68*	1.0005 (0.9979-1.0031), *0.72*	1.0012 (0.9984-1.0041), *0.42*	0.9996 (0.9967-1.0025), *0.8*	0.9991 (0.9960-1.0022), *0.58*
2		1.0085 (0.8382-1.2134), *0.93*	0.9310 (0.7702-1.1254), *0.47*	0.9849 (0.8061-1.2032), *0.89*	2	1.0007 (0.9977-1.0037), *0.66*	0.9991 (0.9961-1.0022), *0.58*	0.9986 (0.9954-1.0018), *0.4*

3			0.9232 (0.7608-1.1203), *0.43*	0.9766 (0.7964-1.1975), *0.83*	3		0.9984 (0.9952-1.0017), *0.34*	0.9979 (0.9945-1.0013), *0.23*
4				1.0578 (0.8590-1.3026), *0.61*	4			0.9995 (0.9960-1.0030), *0.79*
**All-cause infant hospital admissions (52 weeks postnatal), incidence rate**								
1 (most deprived)	0.9857 (0.8478-1.1461), *0.86*	1.0175 (0.8738-1.1848), *0.83*	0.9323 (0.8018-1.0840), *0.37*	1.0444 (0.8743-1.2476), *0.64*	1.0000 (0.9975-1.0026), *1*	1.0018 (0.9992-1.0044), *0.17*	0.9994 (0.9969-1.0020), *0.66*	0.9988 (0.9959-1.0017), 0.43
2		1.0322 (0.8785-1.2128), *0.71*	0.9458 (0.8061-1.1098), *0.5*	1.0595 (0.8801-1.2756), *0.55*	2	1.0018 (0.9991-1.0045), *0.2*	0.9994 (0.9967-1.0021), *0.67*	0.9988 (0.9957-1.0019), *0.45*
3			0.9163 (0.7798-1.0767), *0.29*	1.0265 (0.8516-1.2372), *0.8*	3		0.9976 (0.9949-1.0003), *0.08*	0.9970 (0.9939-1.0001), *0.06*
4				1.1202 (0.9305-1.3487), *0.23*	4			0.9994 (0.9963-1.0025), *0.71*
**Exclusive breastfeeding (10-days postnatal), prevalence**								
1 (most deprived)	1.0536 (0.9655-1.1498), *0.24*	0.9681 (0.8958-1.0462), *0.42*	1.0063 (0.9328-1.0856), *0.88*	1.0029 (0.9317-1.0796), *0.94*	1.0005 (0.9990-1.0020), *0.53*	1.0004 (0.9990-1.0018), *0.57*	0.9997 (0.9984-1.0010), *0.66*	0.9998 (0.9985-1.0011), *0.77*
2		0.9188 (0.8473-0.9964), *0.04*	0.9551 (0.8823-1.0339), *0.26*	0.9519 (0.8811-1.0283), 0.21	2	0.9999 (0.9985-1.0013), *0.9*	0.9992 (0.9979-1.0005), *0.24*	0.9993 (0.9980-1.0006), *0.3*
3			1.0394 (0.9707-1.1130), *0.27*	1.0360 (0.9698-1.1066), *0.3*	3		0.9993 (0.9982-1.0004), *0.23*	0.9994 (0.9983-1.0005), *0.29*
4				0.9967 (0.9350-1.0624), *0.93*	4			1.0001 (0.9991-1.0011), *0.86*
**Exclusive breastfeeding (6-8 weeks postnatal) , prevalence**								
1 (most deprived)	1.0862 (0.9655-1.2220), *0.17*	0.9961 (0.8896-1.1153), *0.95*	1.0228 (0.9182-1.1392), *0.7*	0.9876 (0.8844-1.1027), *0.84*	1.0016 (0.9996-1.0036), *0.12*	1.0012 (0.9993-1.0031), *0.22*	1.0002 (0.9984-1.0020), *0.84*	1.0002 (0.9984-1.0020), *0.84*
2		0.9170 (0.8177-1.0284), *0.14*	0.9416 (0.8440-1.0505), *0.28*	0.9092 (0.8129-1.0168), *0.1*	2	0.9996 (0.9977-1.0016), *0.7*	0.9986 (0.9968-1.0005), *0.14*	0.9986 (0.9968-1.0004), *0.13*
3			1.0268 (0.9251-1.1397), *0.63*	0.9914 (0.8910-1.1032), *0.88*	3		0.9990 (0.9973-1.0007), *0.26*	0.9990 (0.9973-1.0007), *0.25*
4				0.9656	4			1.0000

				(0.8725-1.0685), *0.51*				(0.9984-1.0016), *1*
**Infant sleeping position (6-8 weeks postnatal) , prevalence**								
1 (most deprived)	1.0001 (0.9859-1.0145), *0.99*	1.0022 (0.9870-1.0176), *0.79*	0.9987 (0.9827-1.0150), *0.88*	0.9955 (0.9803-1.0110), *0.58*	1.0000 (0.9998-1.0003), *1*	1.0000 (0.9997-1.0003), *1*	1.0000 (0.9997-1.0003), *1*	1.0001 (0.9998-1.0004), *0.5*
2		1.0021 (0.9865-1.0179), *0.81*	0.9986 (0.9822-1.0153), *0.88*	0.9954 (0.9798-1.0113), *0.58*	2	1.0000 (0.9998-1.0003), *1*	1.0000 (0.9998-1.0003), *1*	1.0001 (0.9998-1.0004), *0.44*
3			0.9965 (0.9793-1.0140), *0.71*	0.9934 (0.9769-1.0101), *0.44*	3		1.0000 (0.9997-1.0003), *1*	1.0001 (0.9998-1.0004), *0.5*
4				0.9968 (0.9795-1.0145), *0.74*	4			1.0001 (0.9998-1.0004), *0.5*
**Infant tobacco smoke exposure (10-days postnatal) , prevalence**								
1 (most deprived)	1.0197 (0.9207-1.1294), *0.72*	0.8857 (0.7808-1.0046), *0.06*	0.8925 (0.7553-1.0546), *0.18*	1.0708 (0.8530-1.3441), *0.57*	0.9995 (0.9976-1.0014), *0.62*	1.0008 (0.9986-1.0031), *0.5*	0.9990 (0.9960-1.0020), *0.52*	1.0009 (0.9972-1.0046), *0.64*
2		0.8685 (0.7607-0.9916), *0.04*	0.8752 (0.7370-1.0394), *0.13*	1.0501 (0.8334-1.3230), *0.69*	2	1.0013 (0.9989-1.0037), *0.29*	0.9995 (0.9964-1.0026), *0.76*	1.0014 (0.9977-1.0052), *0.47*
3			1.0077 (0.8358-1.2150), *0.94*	1.2090 (0.9486-1.5409), *0.13*	3		0.9982 (0.9949-1.0015), *0.29*	1.0001 (0.9962-1.0041), *0.96*
4				1.1998 (0.9194-1.5656), *0.18*	4			1.0019 (0.9975-1.0063), *0.4*
**Infant tobacco smoke exposure (6-8 weeks postnatal) , prevalence**								
1 (most deprived)	1.0843 (0.9141-1.2860), *0.36*	0.9538 (0.7804-1.1657), *0.66*	0.9869 (0.7701-1.2646), *0.92*	1.1140 (0.7941-1.5629), *0.54*	0.9984 (0.9956-1.0012), *0.27*	0.9991 (0.9958-1.0024), *0.61*	0.9990 (0.9949-1.0031), *0.65*	0.9961 (0.9906-1.0017), *0.17*
2		0.8797 (0.7075-1.0938), *0.25*	0.9102 (0.7003-1.1830), *0.49*	1.0274 (0.7247-1.4566), *0.89*	2	1.0007 (0.9971-1.0043), *0.7*	1.0006 (0.9963-1.0049), *0.8*	0.9977 (0.9920-1.0034), *0.44*
3			1.0347 (0.7800-1.3726), *0.82*	1.1680 (0.8111-1.6819), *0.41*	3		0.9999 (0.9952-1.0046), *0.97*	0.9970 (0.9911-1.0030), *0.33*
4				1.1288 (0.7622-1.6717), *0.56*	4			0.9971 (0.9907-1.0036), 0.39
**Primary carer tobacco smoke exposure (10-days postnatal) , prevalence**								

1 (most deprived)	1.0609 (0.9661-1.1651), *0.22*	1.1041 (0.9790-1.452), *0.11*	1.0748 (0.9071-1.2736), *0.41*	1.2237 (0.9953-1.5045), *0.06*	1.0009 (0.9993-1.0025), *0.29*	1.0021 (1.0000-1.0042), *0.05*	1.0024 (0.9994-1.0054), *0.12*	1.0009 (0.9975-1.0043), *0.62*
2		1.0407 (0.9169-1.1812), *0.55*	1.0131 (0.8511-1.2059), *0.89*	1.1534 (0.9346-1.4234), *0.18*	2	1.0012 (0.9990-1.0034), *0.29*	1.0015 (0.9984-1.0047), *0.35*	1.0000 (0.9965-1.0035), *1*
3			0.9735 (0.8051-1.1770), *0.79*	1.1083 (0.8863-1.3858), *0.37*	3		1.0003 (0.9969-1.0037), *0.87*	0.9988 (0.9951-1.0025), *0.54*
4				1.1385 (0.8835-1.4669), *0.32*	4			0.9985 (0.9942-1.0028), *0.51*
**Primary carer tobacco smoke exposure (6-8 weeks postnatal) , prevalence**								
1 (most deprived)	1.0112 (0.8826-1.1585), *0.88*	1.0177 (0.8660-1.1959), *0.84*	0.9352 (0.7561-1.1568), *0.55*	1.2377 (0.9324-1.6430), *0.14*	1.0009 (0.9982-1.0036), *0.53*	1.0009 (0.9977-1.0041), *0.59*	1.0006 (0.9964-1.0048), *0.79*	0.9951 (0.9897-1.0005), *0.08*
2		1.0065 (0.8452-1.1985), *0.95*	0.9249 (0.7401-1.1558), *0.5*	1.2240 (0.9149-1.6375), *0.17*	2	1.0000 (0.9965-1.0035), *1*	0.9997 (0.9953-1.0042), *0.9*	0.9942 (0.9887-0.9998), *0.04*
3			0.9190 (0.7235-1.1673), *0.5*	1.2162 (0.8976-1.6477), *0.21*	3		0.9997 (0.9949-1.0045), *0.91*	0.9942 (0.9884-1.0001), *0.05*
4				1.3234 (0.9478-1.8478), *0.1*	4			0.9945 (0.9881-1.0010), *0.1*

A. Step- and slope-interactions (ratio of Rate Ratios or Prevalence Ratios, 95% Confidence Intervals and p-values) for all Scottish Index of Multiple Deprivation (SIMD) quintiles within each outcome measure. Estimate parameter is Rate Ratio (RR) for measures of incidence rate and Prevalence Ratio (PR) for measures prevalence.

	**Step**<br>**Ratio of RRs (95% CI), *p-value***		**Slope**<br>**Ratio of RRs (95% CI)**	
**Level (age)**	25-38	>38	25-38	>38
**All-cause maternal hospital admissions (26 weeks postnatal), incidence rate**				
<25	1.1557<br>(0.8882-1.5036),<br>*0.28*	1.1312<br>(0.6139-2.0844),<br>*0.71*	0.9995<br>(0.9951-1.0039),<br>*0.84*	0.9957<br>(0.9860-1.0056),<br>*0.4*
25-38		0.9788<br>(0.5402-1.7735),<br>*0.95*	25-38	0.9962<br>(0.9867-1.0058),<br>*0.45*
**All-cause maternal hospital admissions (52 weeks postnatal) , incidence rate**				
<25	1.0631<br>(0.8629-1.3098),<br>*0.58*	1.2318<br>(0.7374-2.0575),<br>*0.43*	1.0017<br>(0.9983-1.0052),<br>*0.34*	0.9962<br>(0.9880-1.0045),<br>*0.38*
25-38		1.1586<br>(0.7050-1.9041),<br>*0.57*	25-38	0.9945<br>(0.9866-1.0026),<br>*0.18*
**All-cause infant hospital admissions (26 weeks postnatal) , incidence rate**				
<25	0.9908<br>(0.8565-1.1462),<br>*0.91*	0.8883<br>(0.6741-1.1707),<br>*0.41*	1.0003<br>(0.9981-1.0025),<br>*0.8*	1.0026<br>(0.9980-1.0072),<br>*0.27*
25-38		0.8965<br>(0.6937-1.1587),<br>*0.41*	25-38	1.0023<br>(0.9980-1.0066),<br>*0.3*
**All-cause infant hospital admissions (52 weeks postnatal) , incidence rate**				
<25	0.9761<br>(0.8582-1.1101),<br>*0.73*	0.9000<br>(0.6734-1.2027),<br>*0.49*	1.0004<br>(0.9982-1.0026),<br>*0.73*	1.0006<br>(0.9957-1.0055),<br>*0.82*
25-38		0.9220<br>(0.6997-1.2150),<br>*0.58*	25-38	1.0002<br>(0.9956-1.0048),<br>*0.94*
**Exclusive breastfeeding (10-days weeks postnatal) , prevalence**				
<25	1.1054<br>(1.0088-1.2112),<br>*0.03*	1.1036<br>(0.9768-1.2468),<br>*0.11*	0.9995<br>(0.9979-1.0011),<br>*0.54*	0.9994<br>(0.9973-1.0015),<br>*0.58*
25-38		0.9984<br>(0.9122-1.0928),<br>*0.97*	25-38	0.9999<br>(0.9984-1.0014),<br>*0.91*

**Exclusive breastfeeding (6-8 weeks postnatal) , prevalence**				
<25	1.1752 (1.0318-1.3386), *0.01*	1.1731 (0.9839-1.3986), *0.07*	0.9993 (0.9971-1.0015), *0.54*	0.9995 (0.9965-1.0025), *0.75*
25-38		0.9982 (0.8752-1.1384), *0.98*	25-38	1.0002 (0.9980-1.0024), *0.87*
**Infant sleeping position (6-8 weeks postnatal) , prevalence**				
<25	1.0049 (0.9913-1.0186), *0.49*	1.0127 (0.9904-1.0355), *0.27*	1.0002 (1.0000-1.0004), *0.08*	1.0003 (0.9999-1.0007), *0.15*
25-38		1.0078 (0.9880-1.0279), *0.45*	25-38	1.0001 (0.9997-1.0005), *0.6*
**Infant tobacco smoke exposure (10-days postnatal) , prevalence**				
<25	1.0110 (0.9256-1.1042), *0.82*	1.0285 (0.8134-1.3005), *0.83*	0.9987 (0.9971-1.0003), *0.12*	1.0006 (0.9964-1.0048), *0.79*
25-38		1.0173 (0.8063-1.2836), *0.89*	25-38	1.0019 (0.9978-1.0061), *0.37*
**Infant tobacco smoke exposure (6-8 weeks postnatal) , prevalence**				
<25	0.9703 (0.8355-1.1268), 0.71	0.7059 (0.4802-1.0377), *0.08*	0.9972 (0.9948-0.9997), *0.03*	0.9981 (0.9918-1.0044), *0.57*
25-38		0.7275 (0.4976-1.0636), *0.1*	25-38	1.0009 (0.9947-1.0071), *0.79*
**Primary carer tobacco smoke exposure (10-days postnatal) , prevalence**				
<25	1.0991 (0.9999-1.2082), *0.05*	1.1108 (0.8679-1.4216), *0.41*	1.0000 (0.9984-1.0016), *1*	0.9987 (0.9946-1.0029), *0.55*
25-38		1.0106 (0.7938-1.2866), *0.94*	25-38	0.9987 (0.9946-1.0028), *0.54*
**Primary carer tobacco smoke exposure (6-8 weeks postnatal) , prevalence**				
<25	1.0751 (0.9557-1.2095), *0.23*	1.1644 (0.8555-1.5849), *0.34*	0.9991 (0.9967-1.0015), *0.46*	0.9995 (0.9934-1.0056), *0.88*

25-38	1.0831 (0.8011-1.4642), *0.62*	25-38	1.0004 (0.9944-1.0064), *0.9*

B. Step- and slope-interactions (ratio of Rate Ratios or Prevalence Ratios, 95% Confidence Intervals and p-values) for all age groupings (<25, 25-38 and >38 years) within each outcome measure. Estimate parameter is Rate Ratio (RR) for measures of incidence rate and Prevalence Ratio (PR) for measures prevalence

Appendix 13

Relative change	
**Step<br>PR (95% CI)**	**Slope<br>PR (95% CI)**
Infant tobacco smoke exposure (10-days postnatal) (s)	
0·8881 (0·8488 , 0·9292)	0·9972 (0·9963, 0·9981)
Infant tobacco smoke exposure (6-8-weeks postnatal)	
0·9763 (0·9057, 1·0525)	1·0010 (0·9995, 1·0025)
Primary carer tobacco smoke exposure (10-days postnatal) (s, d)	
0·9389 (0·8952, 0·9847)	0·9998 (0·9989, 1·0007)

Estimates for truncated models (prevalence Ratio with 95% CI). *s* = adjusted for seasonality and *d* = adjusted for over-dispersion.

Appendix 14

Falsification point	Relative change	
	**Step<br>PR (95% CI)**	**Slope<br>PR (95% CI)**
Infant tobacco smoke exposure (10-days postnatal)		
48 weeks before	1·187 (1·120, 1·259)	1·001 (0·999, 1·003)
24 weeks before	1·034 (0·983, 1·087)	0·998 (0·997, 0·999)
16 weeks before	0·999 (0·950, 1·050)	0·998 (0·997, 0·999)
8 weeks before	0·932 (0·885, 0·981)	0·998 (0·997, 0·999)
Actual	0·904 (0·865, 0·946)	0·998 (0·997, 0·999)
8 weeks after	0·926 (0·879, 0·976)	0·998 (0·998, 0·999)
16 weeks after	0·937 (0·889, 0·987)	0·999 (0·998, 1·000)
24 weeks after	0·987 (0·936, 1·040)	0·998 (0·997, 0·999)
48 weeks after	1·020 (0·959, 1·084)	0·997 (0·996, 0·999)
Primary carer tobacco smoke exposure (10-days postnatal)		
48 weeks before	0·997 (0·944, 1·052)	0·996 (0·994, 0·997)
24 weeks before	0·931 (0·889, 0·976)	0·997 (0·996, 0·998)
16 weeks before	0·914 (0·872, 0·958)	0·997 (0·996, 0·998)
8 weeks before	0·889 (0·848, 0·933)	0·998 (0·997, 0·998)
Actual	0·905 (0·862, 0·950)	0·998 (0·997, 0·999)
8 weeks after	0·886 (0·843, 0·931)	0·999 (0·998, 0·999)
16 weeks after	0·887 (0·844, 0·932)	0·999 (0·998, 1·000)
24 weeks after	0·888 (0·844, 0·935)	1·000 (0·999, 1·001)
48 weeks after	0·972 (0·913, 1·035)	0·999 (0·998, 1·001)
Exclusive breastfeeding - mothers aged <25 years (10-days postnatal)		
48 weeks before	0·862 (0·777, 0·957)	0·997 (0·994, 1·000)
24 weeks before	1·051 (0·952, 1·160)	1·001 (0·999, 1·003)
16 weeks before	1·078 (0·977, 1·188)	1·001 (0·999, 1·002)
8 weeks before	1·104 (1·002, 1·217)	1·000 (0·999, 1·002)
Actual	1·095 (1·004, 1·195)	1·000 (0·998, 1·001)
8 weeks after	1·051 (0·953, 1·159)	1·000 (0·998, 1·001)
16 weeks after	1·045 (0·946, 1·154)	0·999 (0·998, 1·001)
24 weeks after	1·019 (0·920, 1·128)	0·999 (0·997, 1·001)
48 weeks after	1·094 (0·979, 1·221)	0·997 (0·994, 1·000)
Exclusive breastfeeding - mothers aged <25 years (6-8-weeks postnatal)		
48 weeks before	0·803 (0·705, 0·915)	0·995 (0·991, 0·999)

24 weeks before	1·010 (0·892, 1·143)	1·000 (0·998, 1·002)
16 weeks before	1·123 (0·993, 1·270)	1·000 (0·998, 1·003)
8 weeks before	1·119 (0·991, 1·264)	1·000 (0·998, 1·002)
Actual	1·174 (1·037, 1·329)	0·999 (0·997, 1·001)
8 weeks after	1·012 (0·895, 1·143)	0·999 (0·997, 1·001)
16 weeks after	0·993 (0·876, 1·125)	0·999 (0·997, 1·001)
24 weeks after	0·995 (0·875, 1·131)	0·999 (0·997, 1·002)
48 weeks after	1·011 (0·878, 1·162)	0·998 (0·994, 1·002)

Temporal falsification estimates (prevalence ratio with 95% CI) for step- and slope-change at the point of SBBS introduction (actual) and 8, 16, 24 and 48 weeks before and after this point.

Appendix 15

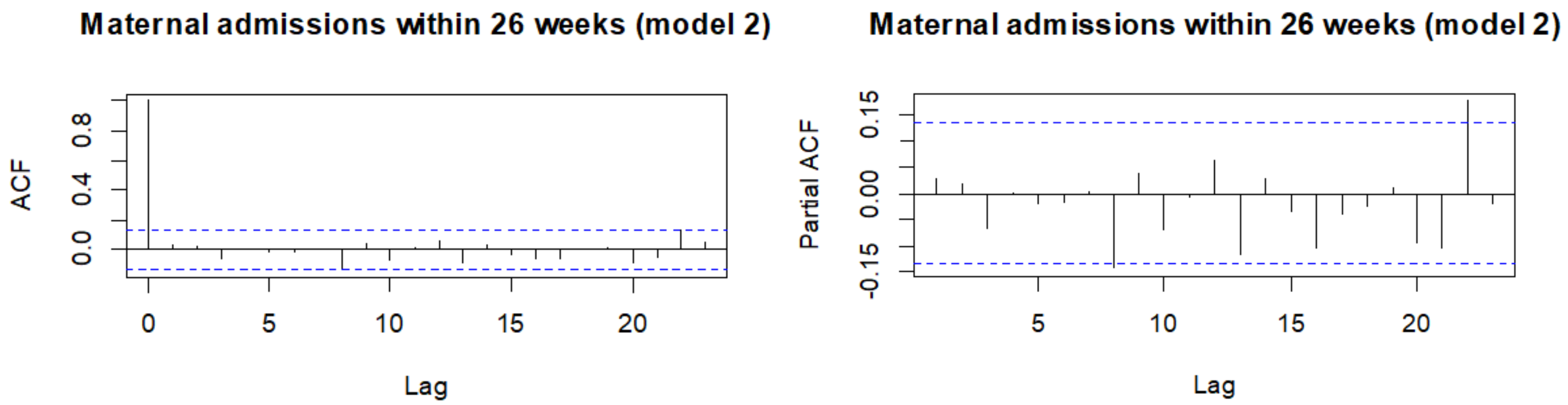

A. Left to right: ACF and P-ACF plots for main analysis of all-cause maternal hospital admissions (26 weeks postnatal), incidence rate x 1000.

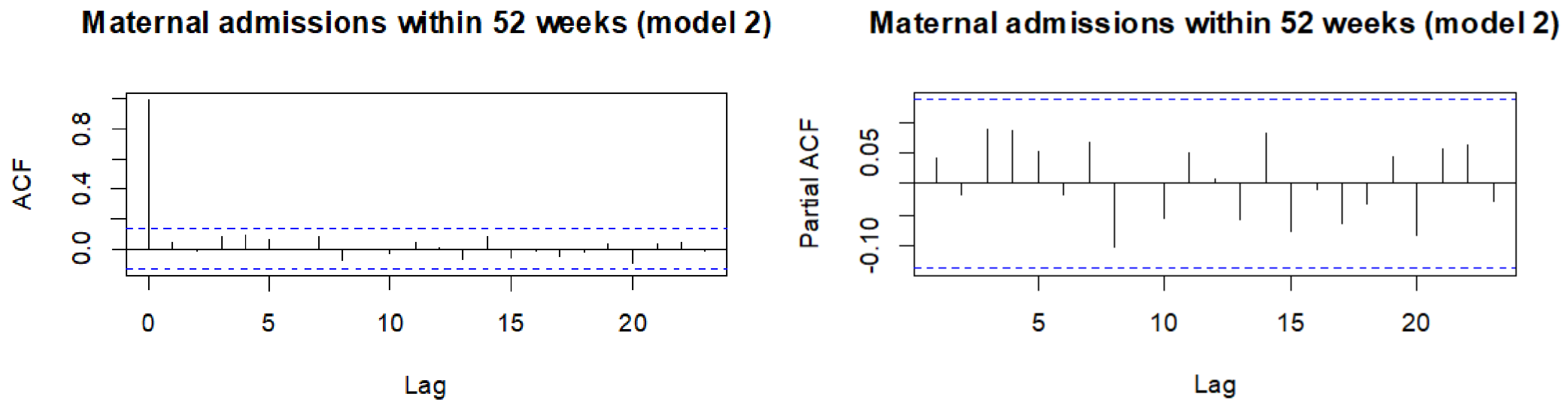

B. Left to right: ACF and P-ACF plots for main analysis of all-cause maternal hospital admissions (52 weeks postnatal), incidence rate x 1000.

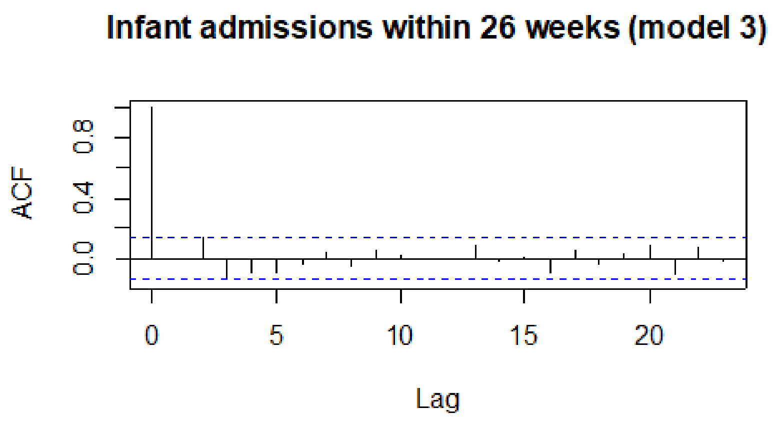

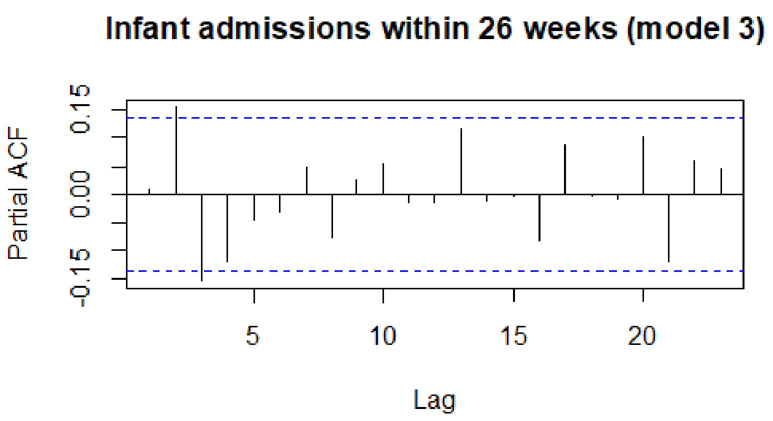

C. Left to right: ACF and P-ACF plots for main analysis of all-cause infant hospital admissions (26 weeks postnatal), incidence rate x 1000.

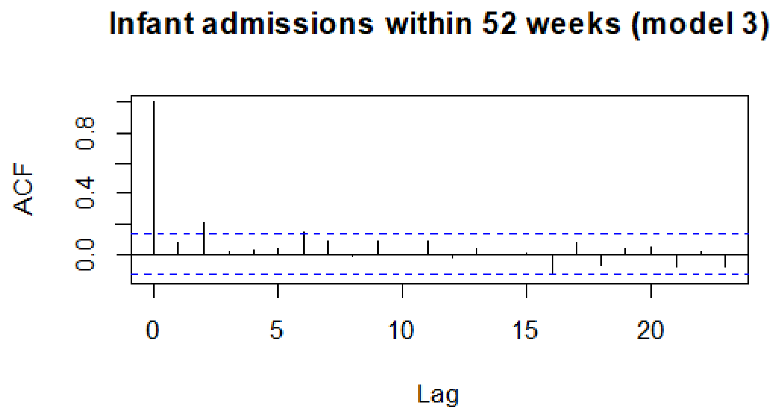

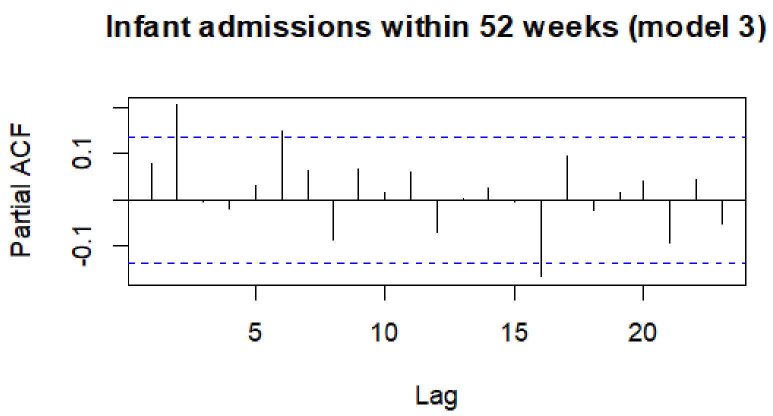

D. Left to right: ACF and P-ACF plots for main analysis of all-cause infant hospital admissions (52 weeks postnatal), incidence rate x 1000.

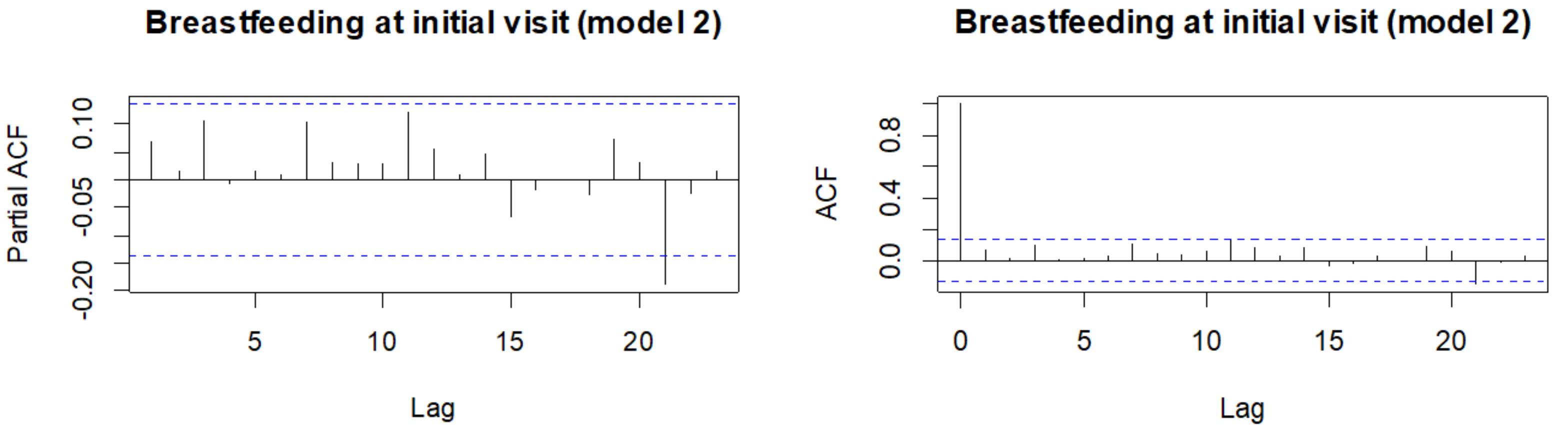

E. Left to right: ACF and P-ACF plots for main analysis of exclusive breastfeeding (10-days postnatal), prevalence.

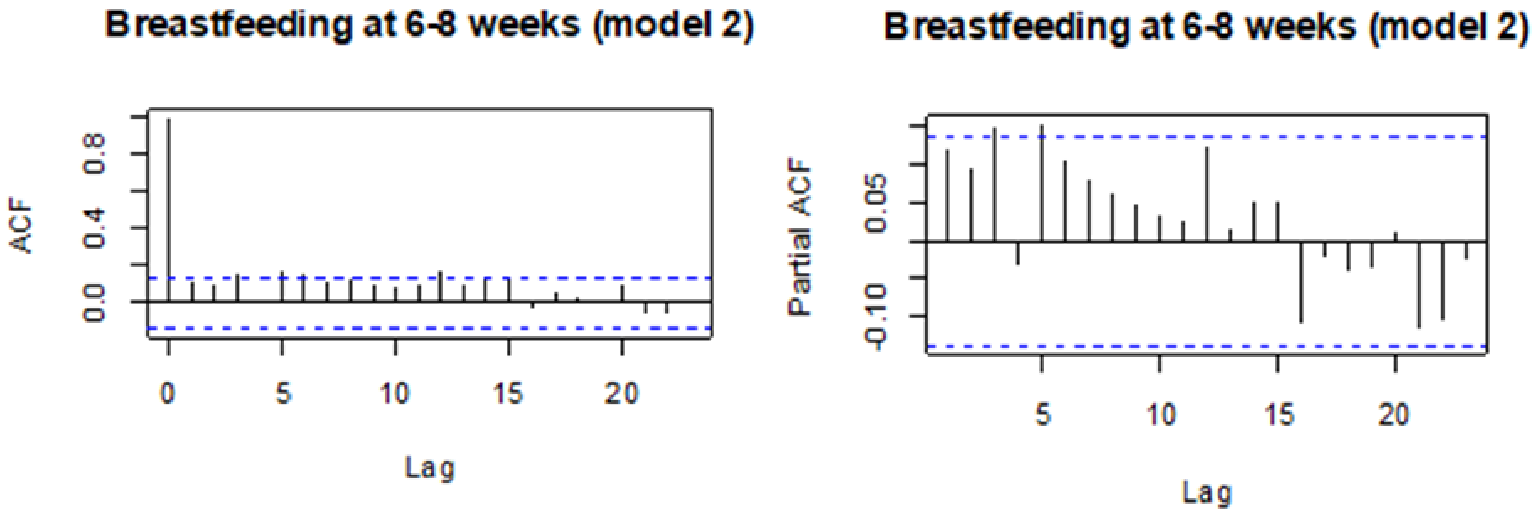

F. Left to right: ACF and P-ACF plots for main analysis of exclusive breastfeeding (6-8-weeks postnatal), prevalence.

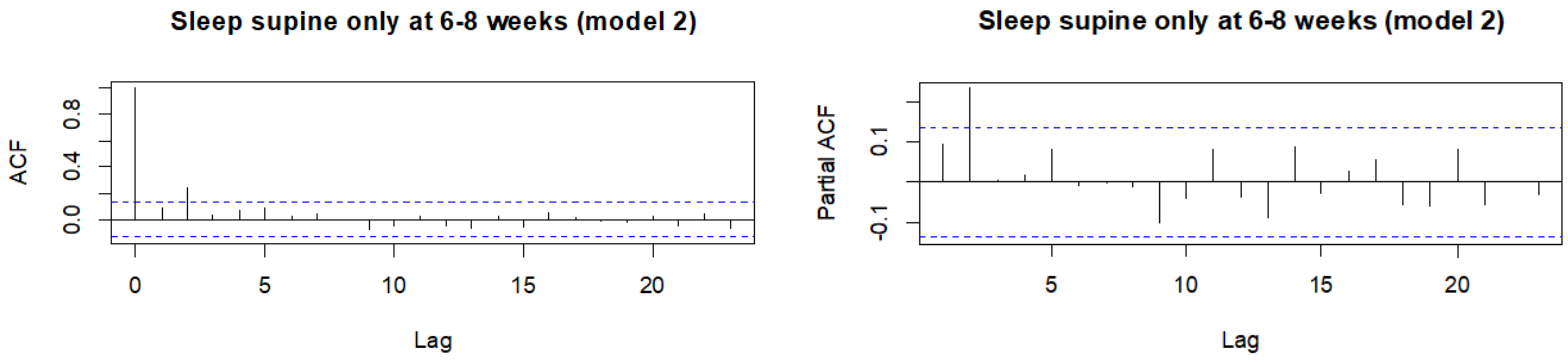

G. Left to right: ACF and P-ACF plots for main analysis of infant sleeping position (6-8 weeks postnatal), prevalence

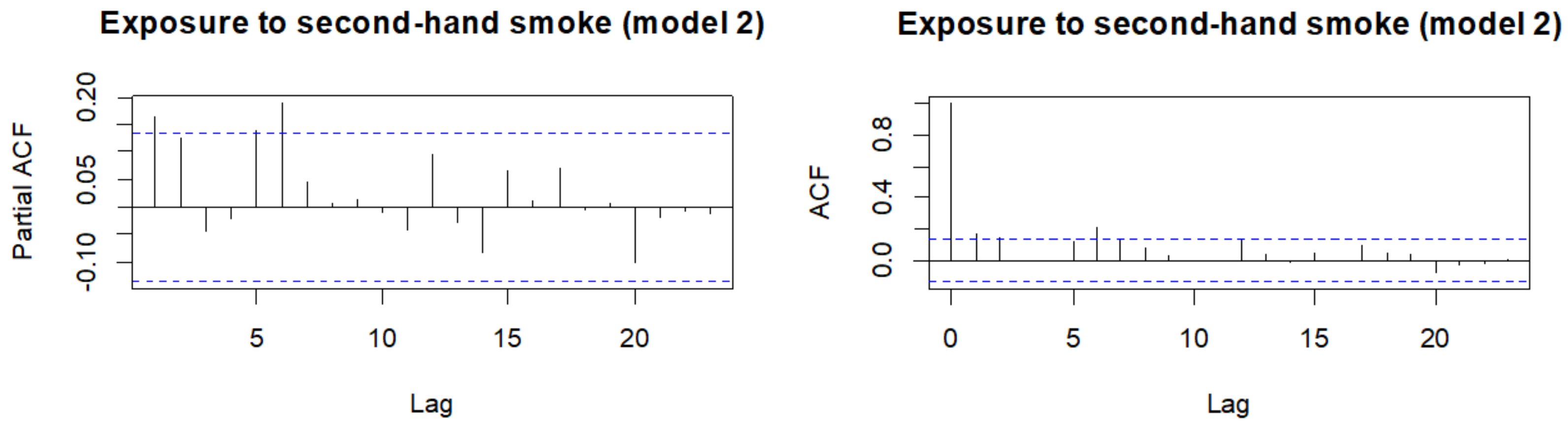

H. Left to right: ACF and P-ACF plots for main analysis of infant exposure to tobacco smoke (10-days postnatal), prevalence

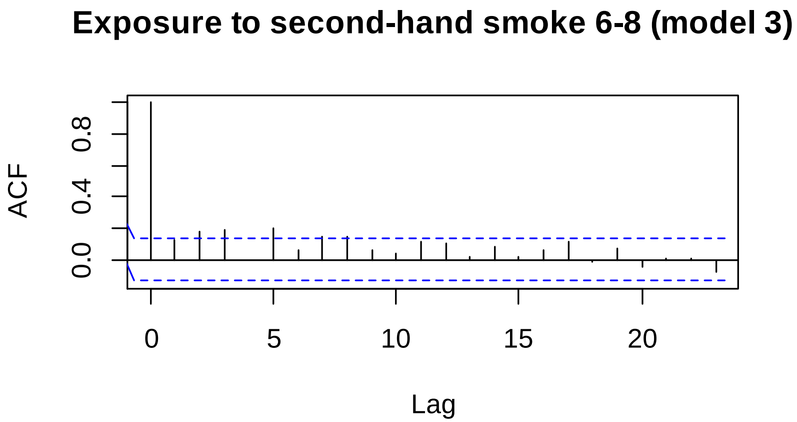

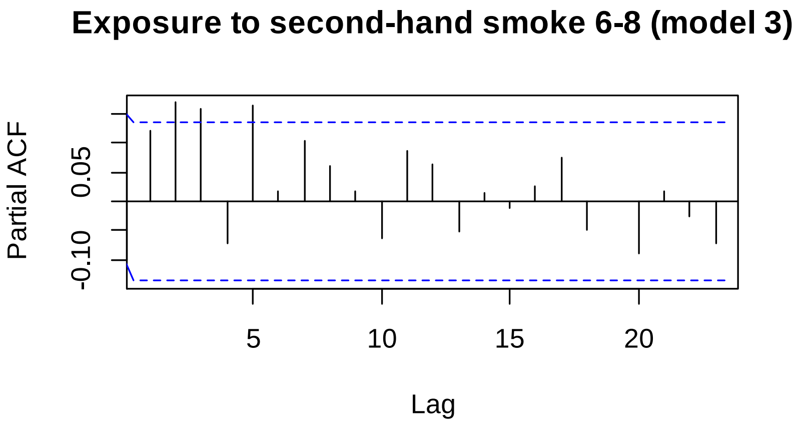

I. Left to right: ACF and P-ACF plots for main analysis of Infant exposure to tobacco smoke (6-8 weeks postnatal), prevalence

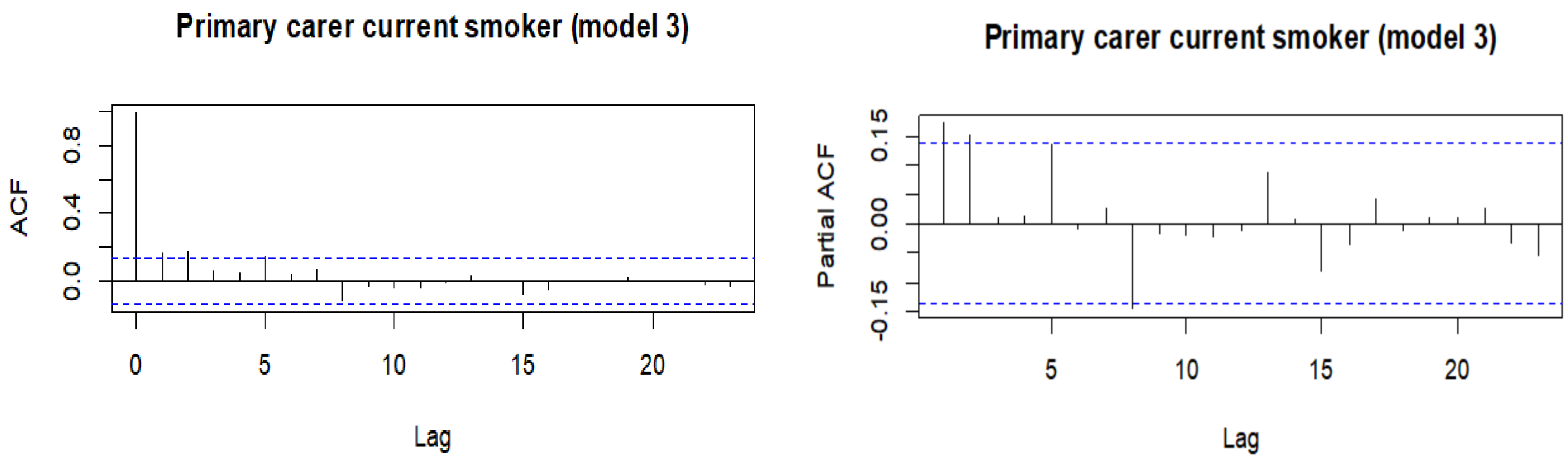

J. Left to right: ACF and P-ACF plots for main analysis primary carer tobacco smoke exposure (10-days postnatal), prevalence

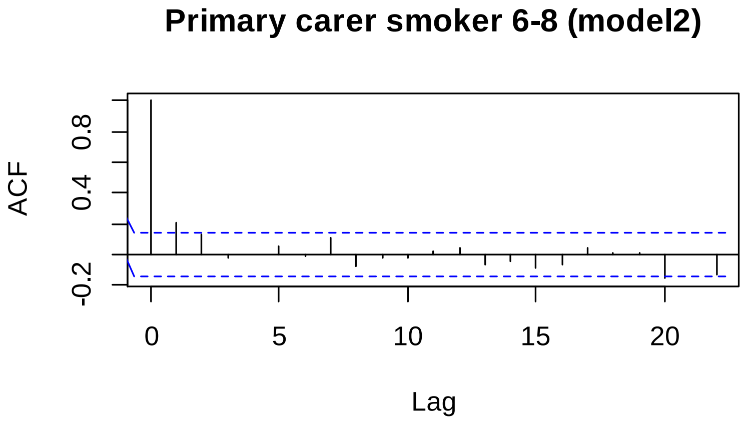

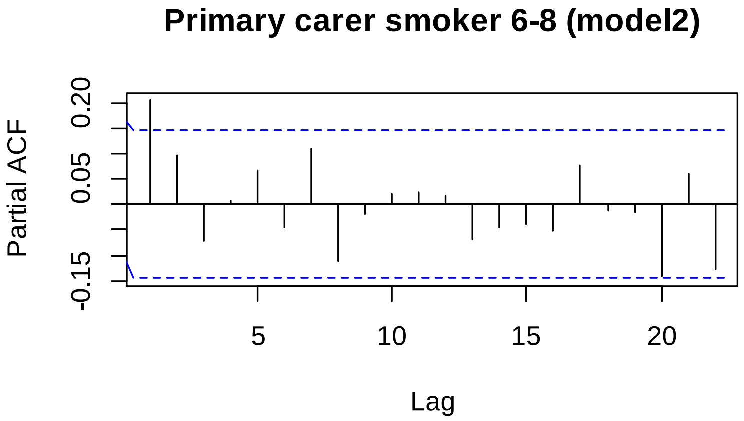

K. Left to right: ACF and P-ACF plots for main analysis primary carer tobacco smoke exposure (6-8 weeks postnatal), prevalence
